# Diagnostic accuracy of cervical cancer screening strategies for high-grade cervical intraepithelial neoplasia (CIN2+/CIN3+) among women living with HIV: A systematic review and meta-analysis

**DOI:** 10.1016/j.eclinm.2022.101645

**Published:** 2022-09-27

**Authors:** Helen Kelly, Iman Jaafar, Michael Chung, Pamela Michelow, Sharon Greene, Howard Strickler, Xianhong Xie, Mark Schiffman, Nathalie Broutet, Philippe Mayaud, Shona Dalal, Marc Arbyn, Silvia de Sanjosé

**Affiliations:** a London School of Hygiene and Tropical Medicine, London, United Kingdom; b National Cancer Institute, National Institutes of Health, Rockville, MD, USA; c Unit of Cancer Epidemiology, Belgian Cancer Centre, Sciensano, Brussels, Belgium; d Department of Global Health, University of Washington, Seattle, USA; e Cytology Unit, Department of Anatomical Pathology, Faculty of Health Science, National Health Laboratory Service, University of the Witwatersrand, Johannesburg, South Africa; f Albert Einstein College of Medicine/Montefiore Medical Center, Bronx, NY, USA; g Department of Global HIV, Hepatitis and STIs Programmes, World Health Organization, Geneva, Switzerland; h Department of Human Structure and Repair, Faculty of Medicine and Health Sciences, University Ghent, Ghent, Belgium; i ISGlobal, Barcelona, Spain

**Keywords:** Cervical cancer, Screening, HIV, HR-HPV, Visual inspection, Cytology, Diagnostic accuracy

## Abstract

**Background:**

We systematically reviewed the diagnostic accuracy of cervical cancer screening and triage strategies in women living with HIV (WLHIV).

**Methods:**

Cochrane Library, Embase, Global Health and Medline were searched for randomised controlled trials, prospective or cross-sectional studies published from database inception to 15 July 2022 reporting diagnostic accuracy of tests in cervical cancer screening and triage of screen-positive WLHIV. Studies were included if they reported the diagnostic accuracy of any cervical cancer screening or triage strategies for the detection of histologically-confirmed high-grade cervical intraepithelial neoplasia (CIN2+/CIN3+) among WLHIV. Summary data were extracted from published reports. Authors were contacted for missing data where applicable. Sensitivity and specificity estimates for CIN2/3+ were pooled using models for meta-analysis of diagnostic accuracy data. Study quality was assessed using the QUADAS-2 tool for the quality assessment of diagnostic accuracy studies. PROSPERO registration:CRD42020189031.

**Findings:**

In 38 studies among 18,737 WLHIV, the majority (n=19) were conducted in sub-Saharan Africa. The pooled prevalence was 12.0% (95%CI:9.8-14.1) for CIN2+ and 6.7% (95%CI:5.0-8.4) for CIN3+. The proportion of screen-positive ranged from 3-31% (visual inspection using acetic acid[VIA]); 2-46% (high-grade squamous intraepithelial lesions, and greater [HSIL+] cytology); 20-64% (high-risk[HR]-HPV DNA). In 14 studies, sensitivity and specificity of VIA were variable limiting the reliability of pooled estimates. In 5 studies where majority had histology-confirmed CIN2+, pooled sensitivity was 56.0% (95%CI:45.4-66.1; *I^2^=65%*) for CIN2+ and 65.0% (95%CI:52.9-75.4; *I^2^*=42%) for CIN3+; specificity for <CIN2 was 73.8% (95%CI:59.8-84.2, *I^2^=94%*). Cytology was similarly variable (sensitivity of ASCUS+ for CIN2+ range: 58-100%; specificity: 9-96%). In 28 studies, sensitivity of tests targeting 14-HR-HPV types was high (91.6%, 95%CI:88.1-94.1; *I^2^=45%* for CIN2+ and 92.5%, 95%CI:88.4-95.2; *I^2^=32%*) for CIN3+); but specificity for <CIN2 was low (62.2% (95%CI:57.9-66.4;*I^2^=92%*). Restriction to 8-HR-HPV increased specificity (65.8%; Relative specificity[RSpec] vs. 14-HR-HPV=1.17; 95%CI:1.10-1.24) with no significant change in sensitivity (CIN2+:85.5%; Relative Sensitivity[RSens]=0.94, 95%CI: 0.89-1.00; CIN3+:90%; RSens=0.96, 95%CI:0.89-1.03). VIA triage of 14-HR-HPV positive women decreased sensitivity for CIN2+ compared to HPV-DNA test alone (64.4% vs. 91.6%; RSens=0.68, 95%CI:0.62-0.75).

**Interpretation:**

HPV-DNA based approaches consistently showed superior sensitivity for CIN2+/CIN3+ compared to VIA or cytology. The low specificity of HPV-DNA based methods targeting up to 14-HR-HPV could be improved significantly by restricting to 8-HR-HPV with only minor losses in sensitivity, limiting requirement for triage for which optimal approaches are less clear.

**Funding:**

World Health Organisation; National Cancer Institute; European Union's Horizon 2020 and Marie Skłodowska-Curie Actions programme.

**Research in context**

**Evidence before this study**

The diagnostic accuracy of cervical cancer screening strategies for CIN2+ detection is well established in the general population, but is less certain for women living with HIV (WLHIV). We conducted a systematic review of the evidence on the diagnostic accuracy of cervical cancer screening strategies for cervical precancer detection in WLHIV. We searched Cochrane Library, Embase, Global Health and Medline databases for studies reporting diagnostic accuracy of any cervical cancer screening strategy to detect cervical precancer (high grade cervical intraepithelial neoplasia [CIN2+/CIN3+]) without language restriction from inception up to 15 July 2022 using search terms “cervical precancer” AND (“HPV-DNA” OR “visual inspection” OR “cytology”) AND “HIV”.

We found that HPV-DNA based tests had superior and more reproducible sensitivity (CIN3+: 93%, 95%CI:88-95; *I^2^=32%*) compared to VIA (65% [95%CI: 53-75, *I^2^*=42%) or cervical cytology (ASCUS+, 89% (95%CI: 81-94, *I^2^*=52%). Despite the lower specificity of HPV-DNA based methods targeting up to 14 high-risk HPV genotypes (<CIN2: 62%, 95%CI: 58-66, *I^2^*=92%), a restricted genotype approach targeting 8 high-risk HPV genotypes most associated with cervical cancer maintained high sensitivity for cervical precancer and increased specificity (66%; Relative specificity vs. 14-HR-HPV=1.18; 95%CI:1.10-1.23). VIA triage of 14-HR-HPV positive women decreased sensitivity compared to HPV-DNA test alone (Relative Sensitivity=0.68, 95%CI:0.62-0.75). Analysis of the sources of study heterogeneity using the QUADAS-2 tool highlighted the inferior performance of the commonly used visual inspection method compared to HPV-DNA test, linked with individual study design and variability in approaches to training and quality assurance for VIA.

**Added value of this study**

To our knowledge, this is the first systematic review and meta-analysis to estimate the diagnostic accuracy of the commonly available cervical cancer screening strategies for CIN2+/CIN3+ detection among WLHIV and to robustly investigate the sources of heterogeneity in these estimates. Our review reports that HPV-DNA based tests have superior and more reproducible sensitivity to detect CIN2+/CIN3+ compared to VIA or cervical cytology. Despite the lower specificity of HPV-DNA based methods targeting up to 14 high-risk HPV genotypes linked to the high prevalence of HR-HPV among WLHIV, a restricted genotype approach targeting 8 high-risk HPV genotypes most associated with cervical cancer maintained high sensitivity for cervical precancer and increased specificity. Such an approach may limit the requirement for triage of screen-positive women in settings where additional clinic visits may prove challenging and for which optimal approaches are less clear.

**Implications of all the available evidence**

Our review supports the WHO guideline recommendations that an HPV DNA based test is the recommended method for screening, irrespective of HIV status, where it is feasible to do so. This review provides a summary and interpretation of all available evidence to date on most widely available screening strategies that may assist decision makers aiming to implement population-based screening approaches to increase screening coverage for the prevention of cervical cancer. Optimal triage options for HPV-positive WLHIV remain unclear. Limitations of existing strategies suggest the need for increased research focus and investment for improved technologies and simple affordable tools that could be made more accessible in settings where cervical cancer incidence and associated mortality are highest.

Alt-text: Unlabelled box

## Introduction

In November 2020, the World Health Organization (WHO) launched a global strategy to eliminate cervical cancer as a public health problem, which means to reduce the annual incidence below 4 cases per 100,000 women.^1^ The targets to achieve elimination rest on three pillars: that 90% of girls are fully vaccinated with the human papillomavirus (HPV) vaccine by age 15, 70% of women are screened with a high-performance test twice between ages 35 and 45, and 90% of women with cervical pre-cancer (i.e. high-grade cervical intraepithelial neoplasia, CIN2+ and CIN3+) or cancer are managed adequately.

Invasive cervical cancer is the second most common cancer and a leading cause of cancer-related death in women living in low- and middle-income countries.^2^ The high mortality from a largely preventable cancer is a consequence of the limited access to HPV vaccination, effective screening or treatment of precancerous lesions in these settings.^3^, ^4^, ^5^ Women living with human immunodeficiency virus (WLHIV) have a 6-fold increased risk of cervical cancer compared to women without HIV.^6^ Of the 19.3 million women living with HIV globally in 2020, 80% were in sub-Saharan Africa where access to cervical cancer screening and treatment of precancerous lesions is limited.

Cervical cancer screening strategies commonly used in resource-limited settings including visual inspection of the cervix or cervical cytology have shown variable diagnostic accuracy to detect cervical precancer due to the requirement for intensive training, equipment and quality assurance.^7^ Clinically validated HR-HPV-DNA tests have high sensitivity to detect precancer with good reproducibility.^8^ However, these tests detect many transient infections, meaning their specificity for cervical precancer is low especially in populations with high prevalence of HR-HPV.^9^ This is problematic among WLHIV who are more likely to have multiple HR-HPV co-infections with a broader range of HR-HPV genotypes^10^ and have a higher risk of HR-HPV incidence and persistence compared to women without HIV.^11^

The diagnostic accuracy of cervical cancer screening strategies for CIN2+ detection is well established in the general population, but is less certain for women living with HIV (WLHIV). Previous reviews have summarised the diagnostic accuracy of cervical cancer screening strategies in WLHIV^12^^,^^13^ although none have yet quantified the pooled sensitivity and specificity to detect precancer in a meta-analysis, evaluated triage strategies of HPV-positive WLHIV, nor conducted a methodological assessment of quality or sources of variability between studies.

In July 2021, the World Health Organization published an update to its guidelines for screening and treatment of cervical pre-cancerous lesions for cervical cancer prevention for women in the general population and women living with HIV. HPV DNA tests were suggested as the primary screening test rather than VIA or cytology for both the general population of women and women living with HIV.^14^ Due to the low specificity of HR-HPV DNA tests, a second test (triage) among HR-HPV positive women is suggested for WLHIV to determine treatment eligibility, although the optimal strategy is not clear. We conducted a systematic review and meta-analysis of the evidence on the diagnostic accuracy of cervical cancer screening strategies for cervical precancer detection in WLHIV which was used to inform the guideline revision.

Our aim was to systematically review and conduct a meta-analysis of the diagnostic accuracy of cervical cancer screening tests for the detection of cervical precancer and above among WLHIV and conduct a structured assessment of study quality and sources of heterogeneity. Screening and triage tests evaluated included visual inspection using acetic acid [VIA], or Lugol's iodine [VILI], cervical cytology and HR-HPV-DNA tests [full and restricted genotyping]). Secondary objectives include evaluation of the modifying effect of HIV-related factors on the diagnostic accuracy of these strategies, and evaluation of the comparative accuracy of HPV-DNA tests compared to VIA or cervical cytology.

## Methods

### Search strategy and selection criteria

We searched the Cochrane Library, Embase, Global Health and Medline databases for publications without language restriction (Appendix, page 2). Reference lists of review articles and all articles identified in the systematic search were checked. The search was conducted up to 15 July 2022, without restriction on start date. All abstracts were screened and assessed for eligibility by one author (HK). Full-text copies of relevant publications were assessed for eligibility independently by two authors (HK, IJ).

Studies were included if they reported the diagnostic accuracy of any cervical cancer screening or triage strategies for the detection of histologically-confirmed CIN2+ or CIN3+ or invasive cervical cancer (reference or gold standard method for outcome ascertainment), irrespective of age at time of screening. Studies were included if there was histological verification of disease among any of: (i) colposcopy abnormal women only; or (ii) screen-positive women only (irrespective of screen test used); or (iii) all enrolled women (i.e., all women underwent colposcopy irrespective of screen test result with directed biopsy and random biopsy of normal quadrants). In studies that corresponded with (i) and (ii), women who did not have biopsy taken because they were either screen negative for one or more tests or colposcopy negative were considered as negative for CIN2+ (i.e. <CIN2).

The index test could include any of the following: VIA (naked eye), VILI, VIA using digital cervicography (VIA-DC), combination VIA/VILI (either test positive), automated or assisted visual evaluation, cervical cytology and any HR-HPV DNA based tests targeting up to 14-HR-HPV types (HPV16/18/31/33/35/39/45/51/52/56/58/59/66/68) using either self-sample or provider-sample. A restricted genotype approach targeting 8-HR types (HPV16/18/31/33/35/45/52/58) was evaluated for tests that provide genotype level data as these types are most commonly associated with invasive cervical cancer, irrespective of HIV status.^15^ Women were considered test-positive if positive for any of the 8-HR types and test-negative if negative for all 8-HR types. Where data were available, the manufacturer defined cut-off to define HPV-DNA test positivity was compared with other possible cut-off values corresponding with difference in HPV viral load^16^ to evaluate impact on test specificity (including variations in relative light unit [RLU] for Hybrid Capture II and PCR-cycle threshold for GeneXpert). For cytology, distinction was made between different threshold for test positivity, including atypical squamous cells of undetermined significance or greater (ASCUS+), low-grade squamous intraepithelial lesion or greater (LSIL+) and high-grade squamous intraepithelial lesion or greater (HSIL+). Co-testing with HR-HPV and cytology (where test-positive occurs when either test is positive and test-negative when both tests are negative) and co-testing with HR-HPV and VIA were also evaluated in context of screening. Index tests were also evaluated in the context of triage following an initial HR-HPV positive test including VIA, cytology (using threshold ASCUS+, LSIL+ or HSIL+) or HPV16/18 genotyping.

Eligible studies could include women with or without HIV, but must have provided data stratified by HIV status. For publications that reported results from the same cohort, but at different follow-up visits, the publication that gave the most complete set of results was included. There was no restriction on age at screening visit. From the consensus list, data were independently extracted by two authors (HK, IJ) using a standardized form. In event of discordance, consensus was reached following detailed discussion with a third author (MA). Authors were contacted for missing data on diagnostic accuracy data where applicable.

### Data analysis

Pooled sensitivity and specificity estimates were obtained using *metadta*, a procedure in Stata implementing bivariate fixed- and random-effects model for pooling of diagnostic test accuracy taking the intrinsic correlation between sensitivity and specificity into account and allowing inclusion of multiple covariates.^17^ The relative sensitivity and specificity of index tests vs comparator test were obtained by including the test as a covariate. The metaregression function of *metadta* was applied to assess the impact of other covariates on diagnostic accuracy (age and HIV-related factors, including ART status and CD4+ T-cell count at time of screening). Heterogeneity was quantified using the I^2^ measure.^18^ Discrete analyses were conducted for each test strategy as: (i) a standalone screening test; (ii) combination of tests, or co-testing and (iii) in triage following an initial HR-HPV positive test. The two former analyses were conducted among women attending routine primary screening, the latter analysis among HR-HPV positive women. Publication bias was assessed using the recommended statistical approach to assess publication bias (or small study size effects) in diagnostic meta-analyses.^19^ The Stata procedure *metaprop* was used to perform meta-analyses of proportions (HR-HPV-positivity, prevalence of CIN2+, CIN3+,proportion receiving ART) in Stata.^20^ As VIA and cytology have been the most frequently used screening modalities in settings where HIV prevalence is highest, relative sensitivity and relative specificity and 95% Confidence Interval (CI) of HPV-DNA based tests (index test) compared to VIA and cervical cytology (comparator tests) was conducted. Only those studies that provided direct head-to-head comparison of those methods in the same women were included. Data were analysed using Stata version 16 (Stata Corporation, College Station. TX: USA).

Study quality was assessed using the QUADAS-2 tool for the quality assessment of diagnostic accuracy studies.^21^ Assessments were conducted as previously reported^22^ (Appendix, page 3-5 for full list of criteria used). The occurrence of partial verification bias and potential gold standard misclassification were key parameters for consideration in the assessment of quality of study methods. Sub-group meta-analyses and metaregresssion were conducted to account for the heterogeneity in methodologies. This review was reported according to the Preferred Reporting Items for Systematic Reviews and Meta-Analyses (PRISMA).^23^ The review protocol is available at https://www.crd.york.ac.uk/PROSPERO/display_record.asp?ID=CRD42020189031.

### Role of the funding source

The funders of the study had no role in the study design, data collection, data analysis, data interpretation, or writing of the report. or decision to submit the paper for publication. HK, IJ and MA had access to and verified the data, and HK was responsible for decision to submit for publication.

## Results

**Figure 1:**
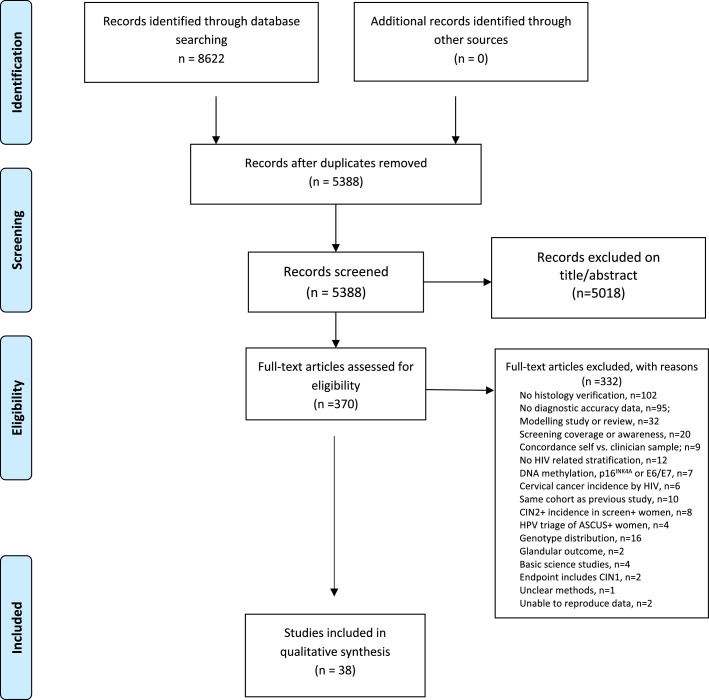
PRISMA Flowchart of included studies. PRISMA= Preferred Reporting Items for Systematic Reviews and Meta-Analyses; HIV=human immunodeficiency virus; DNA= Deoxyribonucleic acid; HPV=human papillomavirus; CIN=cervical intraepithelial neoplasia; CIN2+= cervical intraepithelial neoplasia, grade 2; ASCUS= Atypical squamous cells of undetermined significance.

The search yielded 8,622 publications (Figure 1) among which 38 articles reported the diagnostic accuracy of cervical cancer screening tests for the outcomes CIN2+ or CIN3+, comprising 18,737 WLHIV. Characteristics of individual studies are summarised in Appendix, page 6-8.

The majority of studies were cross-sectional (n=29) and conducted in Sub-Saharan Africa (SSA; n=19; Table 1). Thirteen studies enrolled WLHIV attending cervical cancer screening in outpatient gynaecology clinics,^24^, ^25^, ^26^, ^27^, ^28^, ^29^, ^30^, ^31^, ^32^, ^33^, ^34^, ^35^, ^36^ whereas 21 studies recruited WLHIV attending HIV clinics.^37^, ^38^, ^39^, ^40^, ^41^, ^42^, ^43^, ^44^, ^45^, ^46^, ^47^, ^48^, ^49^, ^50^, ^51^, ^52^, ^53^, ^54^, ^55^, ^56^, ^57^ Two studies recruited WLHIV with a prior positive screen-test (HPV-positive)^58^^,^^59^ and two studies included a combination of women attending primary care and women referred to colposcopy because of positive HPV test and/or abnormal cytology.^60^^,^^61^ The median age of enrolled women within studies ranged from 30 to 50 years (age range: 19 to 68 years). The prevalence of CIN2+ ranged from 2% to 26% (pooled prevalence: 12.0%, 95%CI:9.8-14.1). Pooled CIN3+ prevalence was 6.7% (95%CI: 5.0-8.4).

**Table 1:** Characteristics of included studies.

	All studies	Studies evaluating		
		Visual inspection	Cytology^a^	HPV-DNA
**N studies**	38 24, 25, 26, 27, 28, 29, 30, 31, 32, 33, 34, 35, 36, 37, 38, 39, 40, 41, 42, 43, 44, 45, 46, 47, 48, 49, 50, 51, 52, 53, 54, 55, 56, 57, 58, 59, 60, 61	20 24, 25, 29, 31, 32^,^^36^, ^37^, ^38^, ^39^, ^40^, ^41^, ^42^, ^43^, ^44^, ^45^, ^47^^,^^51^, ^53^^,^^58^^,^^59^	23 25, 26^,^^29^, ^30^^,^^32^^,^^34^, ^36^^,^^37^^,^^39^, ^40^, ^41^, ^42^^,^^45^^,^^46^, ^47^, ^48^^,^^51^, ^52^, ^53^^,^^56^, ^57^, ^58^^,^^61^	28 ^24^^,^^24^, ^27^, ^28^, ^29^, ^30^, ^31^, ^32^, ^33^, ^34^, ^35^, ^36^, ^37^, ^38^, ^39^, ^40^, ^41^, ^42^^,^^45^^,^^46^^,^^49^, ^50^, ^51^^,^^54^, ^55^, ^56^, ^57^^,^^60^^,^^61^
**N women**	18,737	9,781	11,079	15,480
**Study design**				
Cross-sectional	29 24, 25, 26^,^^28^, ^29^, ^30^^,^^33^, ^34^, ^35^^,^^37^, ^38^, ^39^^,^^40^, ^41^, ^42^, ^43^^,^^46^, ^47^, ^48^, ^49^, ^50^, ^51^^,^^53^, ^54^, ^55^, ^56^, ^57^, ^58^, ^59^, ^60^	15 24, 25, 29, 37^,^^38^, ^39^, ^40^, ^41^, ^42^, ^43^, ^47^^,^^51^, ^53^^,^^58^^,^^59^	17 25, 26^,^^29^, ^30^, ^34^^,^^37^^,^^39^, ^40^, ^41^, ^42^^,^^46^, ^47^, ^48^^,^^51^, ^53^^,^^56^, ^57^, ^58^	22 24, 28, 29, 30^,^^33^, ^34^, ^35^^,^^37^, ^38^, ^39^, ^40^, ^41^, ^42^^,^^46^^,^^49^, ^50^, ^51^^,^^54^, ^55^, ^56^, ^57^, ^60^
Prospective cohort	6 27, 32^,^^45^^,^^52^^,^^61^	2 ^32^^,^^45^	5 ^32^^,^^45^^,^^52^^,^^61^	4 27, 45^,^^61^
Randomised controlled trial	2 31, 44	2 31, 44	-	1 ^31^
Retrospective cohort	1 ^36^	1 ^36^	1 ^36^	1 ^36^
**Region**				
Sub-Saharan Africa	19 24, 25, 30, 31, 32, 35, 38, 39^,^^42^, ^43^, ^44^, ^45^, ^47^^,^^49^, ^50^, ^51^^,^^58^, ^59^, ^60^	14 24, 25, 31, 32, 38, 39^,^^42^, ^43^, ^44^, ^45^, ^47^^,^^51^^,^^58^, ^59^	9 25, 30^,^^32^^,^^39^, ^42^, ^45^, ^47^^,^^51^^,^^58^	13 24, 30, 31, 32, 35, 38, 39, 42^,^^45^^,^^49^, ^50^, ^51^^,^^60^
Asia	10 28, 29^,^^33^^,^^36^^,^^37^, ^41^, ^53^, ^54^, ^55^, ^56^	5 29, 36^,^^37^, ^41^, ^53^	6 29, 36^,^^37^, ^41^, ^53^^,^^56^	9 28, 29^,^^33^^,^^36^^,^^37^, ^41^^,^^54^, ^55^, ^56^
Latin America	2 27, 34	-	1 ^34^	2 27, 34
North America	4 ^40^^,^^48^^,^^57^^,^^61^	1 ^40^	4 ^40^^,^^48^^,^^57^^,^^61^	3 ^40^^,^^57^^,^^61^
Europe a	3^26^^,^^46^^,^^52^	-	3 ^26^^,^^46^^,^^52^	1 ^46^
**Enrolment period**^b^		-		
Pre-combination ART (pre-1996)	3 ^40^^,^^48^^,^^52^	1 ^40^	3 ^40^^,^^48^^,^^52^	1 ^40^
Early ART (1996-2008)	8 26, 27, 31, 34, 35^,^^46^^,^^50^, ^53^	2 31, 53	4 26, 34^,^^46^, ^53^	6 27, 31, 34, 35, 46^,^^50^^,^
Recent ART (2009-2015)	19 24, 25, 28, 29, 30^,^^36^, ^37^, ^38^, ^39^, ^42^, ^43^, ^44^, ^45^^,^^49^^,^^54^, ^55^, ^56^^,^^58^^,^^61^	12 24, 25, 29, 36^,^^37^, ^38^, ^39^^,^^42^, ^43^, ^44^, ^45^^,^^58^	11 25, 29, 30^,^^36^^,^^37^, ^39^, ^42^^,^^45^^,^^56^^,^^58^^,^^61^	15 24, 28, 29, 30^,^^36^, ^37^, ^38^, ^39^, ^42^^,^^45^^,^^49^^,^^54^, ^55^, ^56^^,^^61^
Universal ART (post-2015)	5 32, 41^,^^51^^,^^59^^,^^60^	4 32, 41^,^^51^^,^^59^	3 32, 41^,^^51^	4 32, 51^,^^60^
Not reported	3 33, 47^,^^57^	1 ^47^	2 47, 57	2 ^33^^,^^57^
**Median CD4+ count, cells/µl (range)**	271-592	271-550	271-592	347-592
**Taking ART, %**^c^	72.8% (65.8-79.7)	72.9 (64.1-81.7)	75.2 (66.3-84.1)	75.5 (67.9-83.1)
**Median Age, years (range)**	30-50	30-46	30-50	32-50
**HR-HPV pooled prevalence, % (IQR)**^d^	45.0% (38.8-51.1)	44.2% (35.3-53.2)	44.1% (35.8-52.5)	45.0% (38.8-51.1)
**CIN2+ pooled prevalence, % (IQR)**^d^	12.0% (9.8-14.1)	12.8% (9.5-16.1)	13.2% (10.1-16.3)	11.0% (8.6-13.4)
**CIN3+ pooled prevalence, % (IQR)**^d^	6.7% (5.0-8.4)	6.5% (4.6-8.3)	7.3 % (5.2-9.4)	6.2% (4.3-8.2)

a All studies used conventional cytology (Papanicolaou method), except one study which used liquid based cytology (LBC)^51^ and one multi-country study in which there was a mix of conventional and LBC.^46^

b Pre-combination ART (pre-1996); early ART (1996-2008); recent ART (2009-2015); post-combination ART (1996-2015); universal ART (post-2015); IQR=interquartile range; one study^60^ enrolled a combination of women attending primary health care (62% of all women enrolled) and women referred to colposcopy clinics due to abnormal cytology (38% of all women); CIN2+ prevalence was 15.3% in women from primary health care and 55.4% in women from colposcopy clinics and 31.5% overall.

c ART status not reported for 9 studies.

d Studies enrolling WLHIV with a prior positive screen-test (HPV-positive)^58^^,^^59^ and two studies included a combination of women attending primary care and women referred to colposcopy because of positive HPV test and/or abnormal cytology.^60^^,^^61^ excluded from pooled estimates.

The majority of studies recruited women in the post-combination ART era (post-1996) when guidelines recommended initiation of ART at defined CD4+ T-cell count, but before the universal ART era (pre-2015) when ART is recommended at time of HIV diagnosis, irrespective of CD4+ T-cell count. The median CD4+ T-cell count ranged from 271 to 592 cells/µl. No studies reported prior HPV vaccination of enrolled WLHIV.

Twenty studies^24^, ^25^, ^29^, ^31^, ^32^, ^36^, ^37^, ^38^, ^39^, ^40^, ^41^, ^42^, ^43^, ^44^, ^45^, ^47^, ^51^, ^53^, ^58^, ^59^ evaluated any visual inspection strategy (VIA, VIA-DC or VILI) for CIN2+ detection (Table 1) and 12 for CIN3+^29^, ^36^, ^37^, ^38^, ^39^^,^ 42, 45, 47^,^^51^^,^[53] the majority were conducted in SSA. No studies evaluated automated visual evaluation (AVE) methods. Twenty-three studies evaluated cervical cytology for CIN2+: 18, 13 and 16 using threshold ASCUS+,^25^, ^26^, ^29^^,^^32^^,^^36^^,^^37^, ^39^^,^^40^, ^41^, ^42^^,^^45^, ^46^, ^47^, ^48^^,^^51^, ^53^^,^^56^^,^^57^ LSIL+^25^, ^29^, ^34^, ^36^^,^ 37, 39, 42^,^^45^, ^46^, ^47^, ^48^^,^^51^, ^53^ and HSIL+^25^, ^26^^,^^29^, ^30^^,^^32^, ^34^^,^^36^^,^^37^^,^^39^, ^42^, ^45^, ^47^^,^ ^48^^,^^51^^,^^52^, ^53^, respectively; 14 for CIN3+.^25^, ^29^, ^30^^,^^32^^,^^36^^,^ ^37^^,^^39^, ^41^, ^42^, ^45^^,^^47^, ^48^^,^^51^, ^53^. Twenty-eight studies evaluated any HR-HPV DNA test method for CIN2+^24^, ^27^, ^28^, ^29^, ^30^, ^31^, ^32^, ^33^, ^34^, ^35^, ^36^, ^37^, ^38^, ^39^, ^40^, ^41^, ^42^^,^^45^^,^^46^^,^^49^, ^50^, ^51^^,^^54^, ^55^, ^56^, ^57^^,^^60^ and 18 for CIN3+;^25^, ^28^, ^29^, ^30^^,^^33^^,^^36^^,^^37^, ^39^, ^41^, ^42^^,^ 45, 47^,^^49^, ^50^, ^51^, ^53^^,^^60^^,^^61^ these included Hybrid Capture II (HC-II; 16 studies);^27^, ^29^, ^31^, ^34^, ^35^, ^36^^,^^37^^,^^40^, ^41^, ^42^^,^^45^^,^^46^^,^^50^^,^^54^, ^55^^,^^57^ GeneXpert (4 studies);^32^, ^38^, ^49^^,^^60^ careHPV (3 studies)^24^, ^45^^,^^56^ and GP5+/6+ PCR (2 studies^30^, ^39^). The proportion of screen-positive WLHIV ranged from 3-31% (VIA), 2-46% (HSIL+ cytology), and 20-64% (HR-HPV DNA).

The range of quality judgements is summarised for 12 QUADAS items in Appendix, pages 9-14. For 14 studies evaluating VIA, a good quality score was given in 64% (108 of the 14*12 judgements). Low and unclear study quality scores (QUADAS score=N and U, respectively) were noted in 16% and 20% of the judgements. In 23 studies evaluating cervical cytology, the corresponding proportions were 54%, 23% and 23% for high, low and unclear, respectively. In 28 studies evaluating HPV-DNA, the corresponding proportions were 64%, 18% and 18% for high, low and unclear, respectively. For studies evaluating VIA and cytology, the study quality was judged as low for the following items: quality control of index test, misclassification of disease avoided, partial verification avoided and representativeness of the included participants. For studies evaluating HPV-DNA tests, the study quality was judged as low for items related to misclassification of disease, partial verification and representativeness of the included participants.

**Figure 2:**
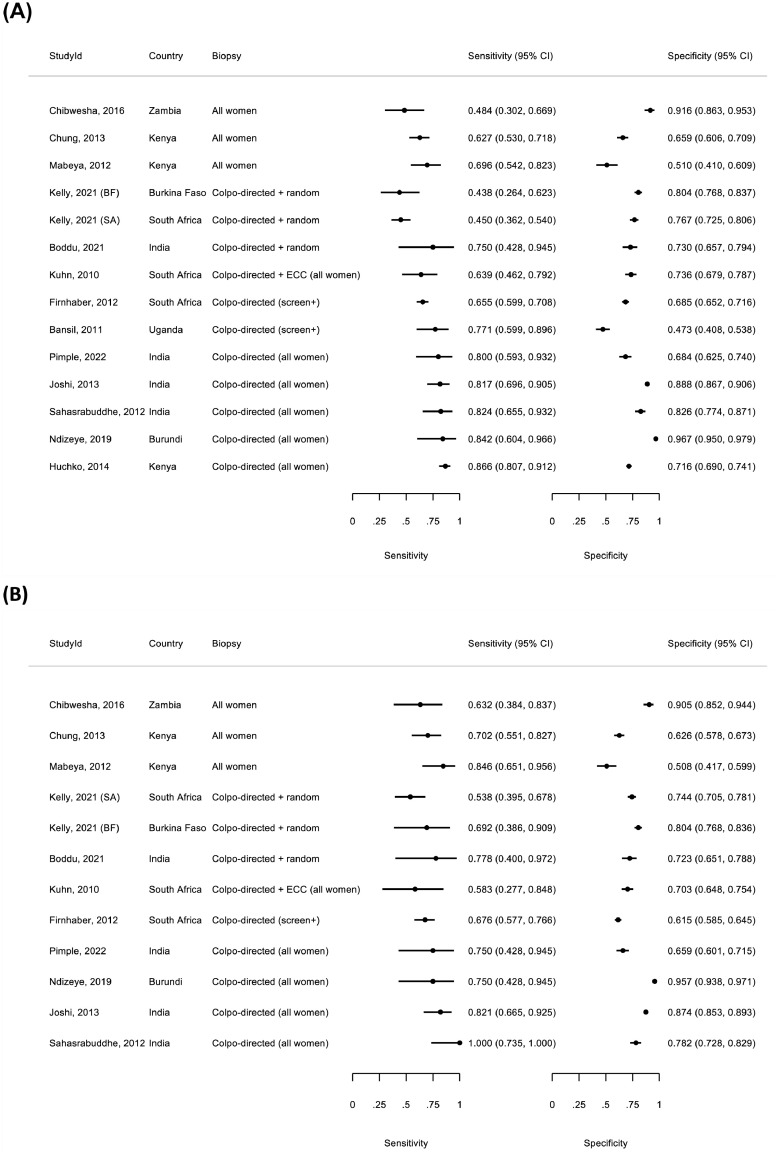
**Forest plot of diagnostic accuracy of VIA for CIN2+ (A) in 14 populations and CIN3+ (B) in 12 populations of WLHIV**. Pooled estimates not calculated for VIA given the heterogeneity in sensitivity and specificity across studies. Stratified pooled sensitivity and specificity according to proportion of women undergoing histology verification are given in Tables 2 and 3. *Biopsy and histological verification of disease varied between studies but four approaches to biopsy indication and histology verification were identified: (i) all women underwent colposcopy; biopsy was taken of abnormal areas plus random biopsy of normal quadrants (i.e. all enrolled women had biopsy and histology verification)^31^^,^^38^^,^^39^^,^^45^^,^^47^; (ii) all women underwent colposcopy; women who were screen test positive for any of HPV-DNA, cytology (ASCUS+) or VIA/VILI abnormal or colposcopy abnormal underwent biopsy of abnormal areas plus random biopsy of normal quadrants^37^^,^^45^; (iii) all women underwent colposcopy; colposcopy directed (colpo-directed) biopsy was taken of abnormal areas^24^^,^^36^^,^^42^; (iv) women who screen test positive (single or multiple screening tests) were referred to colposcopy, colposcopy directed biopsy was taken of abnormal areas.^29^^,^^43^^,^^51^^,^^53^ The proportion of women with histology verification decreased from category (i) to (iv). VIA=visual inspection using acetic acid; VILI=visual inspection using lugol's iodine; ECC=endocervical curettage; ASCUS= Atypical squamous cells of undetermined significance; CIN2+= cervical intraepithelial neoplasia, grade 2; CIN3+= cervical intraepithelial neoplasia, grade 3; WLHIV=women living with HIV; Kelly, 2021 (BF) and Kelly, 202 (SA) refers to diagnostic accuracy estimates among women included in the Burkina Faso and South Africa sites, respectively; CI=confidence intervals.

In 14 studies,^24^^,^^29^^,^^31^^,^^36^, ^37^, ^38^, ^39^^,^^42^^,^^43^^,^^45^^,^^47^^,^^51^^,^^53^^,^^51^ the sensitivity of VIA for CIN2+ ranged from 43.8% to 86.6% and specificity for <CIN2 from 47.3% to 96.7%. Figure 2 shows the wide variation in sensitivity and specificity for CIN2+ /CIN3+. Diagnostic accuracy estimates varied according to different approaches to gold standard verification. In five studies where the majority (>95%) of WLHIV underwent biopsy and histological verification and thus had the lowest risk of incomplete verification,^31^, ^38^, ^39^, ^45^, ^47^ VIA had pooled sensitivity of 56.0% (95%CI: 45.4-66.1; *I^2^*=65%; Table 2) for CIN2+. Sensitivity was higher in studies with a greater proportion of WLHIV with incomplete verification (Table 2). In a small number of studies, sensitivity of VILI and VIA-DC for CIN2+/CIN3+ was higher than VIA alone (Appendix, page 15).

**Table 2:** Meta-analysis of diagnostic accuracy of cervical cancer screening strategies for CIN2+ among WLHIV.

	N studies*	N WLHIV	CIN2+ prevalence, % (95%CI)	Test positive, % (95%CI)	Sensitivity for CIN2+ (%, 95%CI)	*I**^2^*	Specificity for <CIN2 (%, 95%CI)	*I**^2^*	p-value**
**Screen approach**									
VIA – naked eye^a^									
Studies with ≥95% histology verification 31, 38, 39, 45, 47	5	1703	20.6 (15.5-26.6)	33.7 (22.7-44.7)	56.0 (45.4-66.1)	*64.9%*	73.8 (59.8-84.2)	*94.4%*	*0.24*
Studies with 50-95% histology verification 24, 37^,^^42^, ^45^	4	1932	12.8 (2.2-23.3)	36.7 (23.0-50.5)	65.1 (52.1-76.1)	*58.5%*	68.3 (55.6-78.8)	*94.7%*	*0.97*
Studies with <50% histology verification ^29^^,^^43^^,^^51^^,^^53^	5	4090	8.1 (4.1-12.0)	23.3 (10.7-36.0)	83.9 (78.6-88.2)	*5.5%*	85.0 (71.5-92.7	*97.2%*	*0.44*
***Cytology***									
Cytology ASCUS+ 25, 26, 29^,^^32^^,^^36^^,^^37^^,^^39^, ^40^, ^41^, ^42^, ^45^, ^46^, ^47^, ^48^^,^^51^, ^53^^,^^56^^,^^57^	19	8916	12.2 (9.1-15.4)	40.6 (25.2-56.0)	85.1 (78.1-90.1)	*67.6%*	68.3 (55.7-78.6)	*97.4%*	*0.32*
Cytology LSIL+ 25, 29, 36^,^^37^, ^39^, ^41^, ^42^^,^^45^^,^^46^, ^47^^,^^51^, ^53^	14	7539	12.8 (9.0-16.6)	34.5 (18.3-50.7)	80.9 (72.5-87.3)	*80.7%*	75.6 (64.2-84.0)	*98.0%*	*0.40*
Cytology HSIL+ 25, 26, 29^,^^30^^,^^32^, ^34^, ^36^, ^39^^,^^42^, ^45^, ^47^^,^^48^^,^^51^, ^52^, ^53^	17	6852	15.0 (10.7-19.2)	14.2 (9.4-19.0)	44.5 (33.7-55.8)	*75.9%*	96.3 (93.8-97.8)	*84.8%*	*0.93*
***HPV based tests***									
*All*^27^, ^28^, ^29^, ^30^, ^31^, ^32^, ^33^, ^34^, ^35^, ^36^, ^37^, ^38^, ^39^, ^40^, ^41^, ^42^^,^^45^^,^^46^^,^^49^, ^50^, ^51^^,^^54^, ^55^, ^56^, ^57^^,^^,^^60^	28	14628	12.6 (9.8-15.5)	44.7 (39.2-50.1)	91.6 (88.1-94.1)	*44.9%*	62.2 (57.9-66.4)	*92.2%*	*0.27*
Hybrid Capture II^b^^27^, ^29^, ^31^, ^34^, ^35^, ^36^, ^37^^,^^40^, ^41^, ^42^^,^^45^^,^^46^^,^^50^^,^^54^, ^55^^,^^57^	17	9124	10.1 (7.3-12.9)	46.0 (39.5-52.5)	94.2 (91.3-96.2)	*18.6%*	59.4 (53.8-64.8)	*91.2%*	*0.73*
GeneXpert^c^^32^, ^38^, ^49^^,^^60^	4	2036	23.2 (11.3-35.1)	49.7 (34.4-65.0)	93.0 (87.1-96.3)	*41.1%*	62.6 (50.3-73.5)	*93.7%*	*0.29*
CareHPV^d^^24^, ^45^^,^^56^	4	2012	7.7 (2.4-13.1)	41.4 (36.6-46.2)	92.3 (81.0-97.1)	*27.3%*	62.8 (58.4-67.0)	*67.4%*	*0.92*
GP5+/6+ 30, 39	2	738	26.9 (23.7-30.1)	50.0 (46.4-53.6)	81.1 (75.1-85.9)	*-*	61.6 (57.5-65.7)	*-*	*-*
Restricted genotyping***									
8-HR [low threshold]^e^^39^, ^45^^,^^60^	4	2018	22.7 (8.3-37.2)	45.4 (34.9-55.9)	85.5 (75.3-91.9)	*72.2%*	65.8 (60.0-71.1)	*81.0%*	*0.55*
8-HR [high threshold]^e^^45^^,^^60^	3	1651	22.2 (3.5-40.8)	43.5 (31.2-55.7)	83.5 (69.0-92.0)	*75.6%*	76.6 (71.8-80.9)	*71.4%*	*0.52*
OncoE6 (HPV16/18/45) 38, 51	2	879	3.5 (2.3-4.7)	3.4 (2.2-4.6)	35.3 (23.5-49.2)	*-*	98.3 (97.2-99.0)	*-*	*-*
**Co-testing (either test positive)^f^**									
HPV DNA or VIA positive 29, 39, 42, 45	5	3896	16.7 (7.5-25.9)	57.8 (42.2-73.1)	95.4 (90.5-97.8)	43.6%	48.6 (37.9-59.4)	94.3%	0.61
HPV DNA or HSIL+ 39, 42, 45	4	2752	19.6 (8.5-30.8)	56.8 (48.0-65.6)	95.5 (90.5-97.9)	3.4%	56.8 (45.8-67.1)	73.6%	0.49
**Triage of HPV positive WLHIV**^g^									
HPV -> VIA 32, 42^,^^45^^,^^51^^,^^58^^,^^59^	7	2216	26.0 (14.3-37.6)	35.4 (18.7-52.1)	57.4 (41.8-71.7)	57.8%	79.9 (64.4-89.7)	90.6%	0.46
HPV -> HPV16/18 ^45^^,^^51^^,^^58^	4	1073	22.6 (8.4-36.7)	23.9 (10.9-36.8)	38.3 (25.9-52.4)	0.8%	80.4 (66.1-89.7)	86.3%	0.05
HPV -> cytology ASCUS+ ^32^^,^^45^^,^^58^	4	1124	29.7 (16.9-42.5)	60.9 (30.9-90.9)	91.7 (83.9-95.9)	21.6%	49.8 (30.8-68.8)	93.8%	0.03
HPV -> cytology LSIL+ ^45^^,^^58^	3	1042	28.0 (12.8-43.3)	62.1 (26.6-97.6)	90.8 (83.2-95.2)	60.5%	48.8 (31.2-66.8)	95.8%	0.84
HPV -> cytology HSIL+ ^32^^,^^42^, ^45^^,^^58^	5	1838	31.8 (20.9-42.8)	29.8 (12.1-47.5)	63.2 (47.4-76.7)	80.9%	91.1 (82.1-95.8)	81.4%	0.94

*one study^45^ contributed diagnostic accuracy for two distinct populations in two countries; each country specific estimate is considered as a seperate study in the meta-analysis; **p-value for publication bias; ^a^conducted by nurse/midwife; ^b^HC-II targets 13 HR types: HPV-16, -18, -31, -33, -35, -39, -45, -51, -52, -56, -58, -59, and -68; ^c^GeneXpert 5-channel targets 14 HR types: HPV-16, -18, -31, -33, -35, -39, -45, -51, -52, -56, -58, -59, 66 and -68; ^d^CareHPV targets 14 HR types: HPV-16, -18, -31, -33, -35, -39, -45, -51, -52, -56, -58, -59, -66 and -68; ^e^8 HR types: HPV 16; HPV 18 or 45; or HPV 31, 33, 35, 52, or 58; analysis includes two studies used HC-II and INNO-LiPA (positive for HC-II and any HPV16/18/45/31/33/35/52/58; low threshold=1RLU; high threshold=20RLU)^35^, ^45^ and one study used GeneXpert (low threshold=a higher number of replication cycles; high threshold=low number of replication cycles)^60^; ^f^screen positive when either test was positive and screen negative when both tests were negative, all women receive both tests; ^g^tests conducted among HPV positive women only; ***Restricted genotype - One study used GeneXpert^60^; one study among WLHIV in Kenya used GP5+/6+ PCR^39^ and one study in two populations of WLHIV in South Africa and Burkina Faso used INNO-LIPA genotyping assay.^45^ For the latter study, test positivity was defined as positivity for any of those genotypes among women who were also HC-II positive because of the low limit of detection of INNO-LiPA and to improve clinical relevancy.

**Figure 3:**
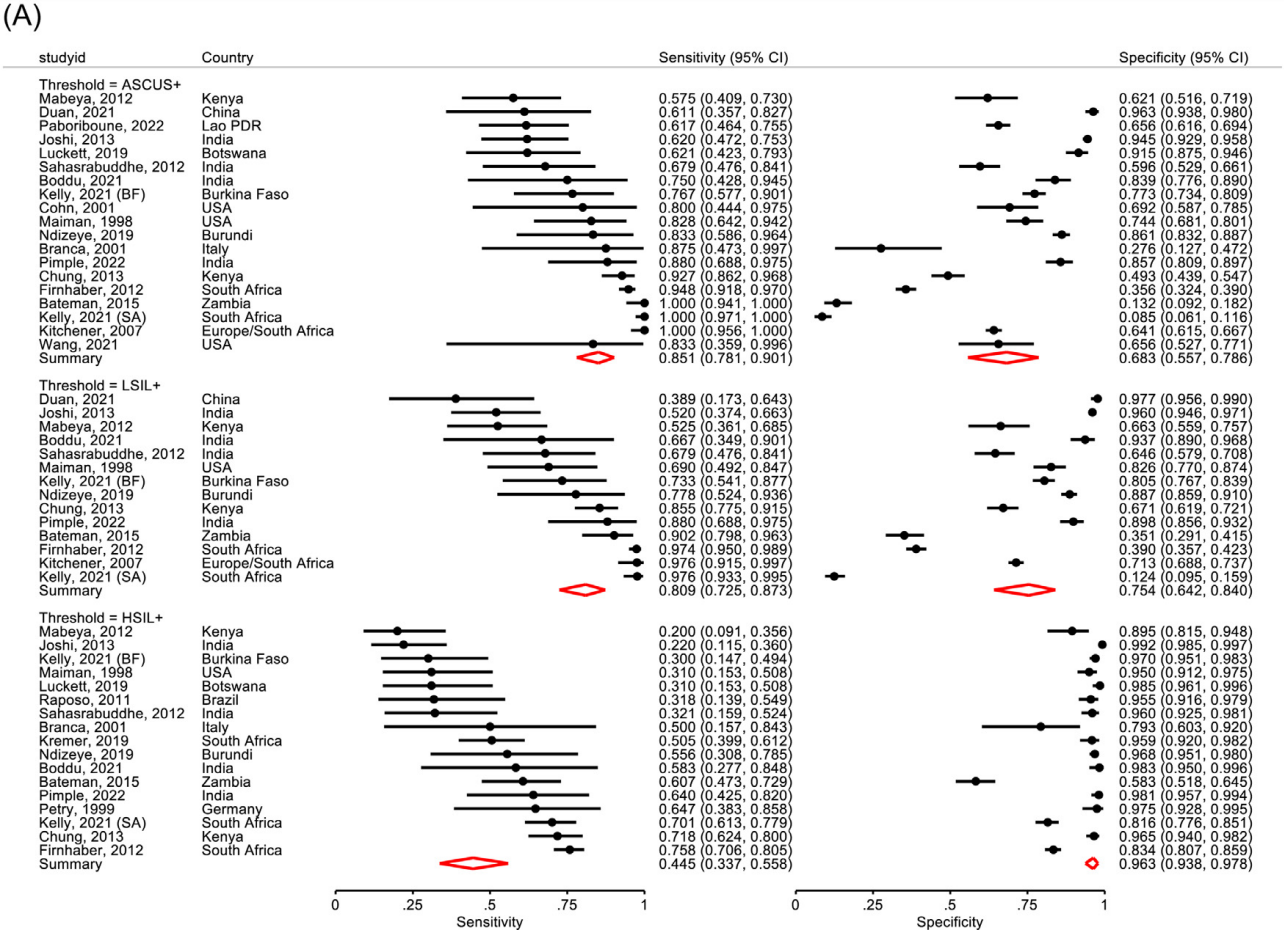

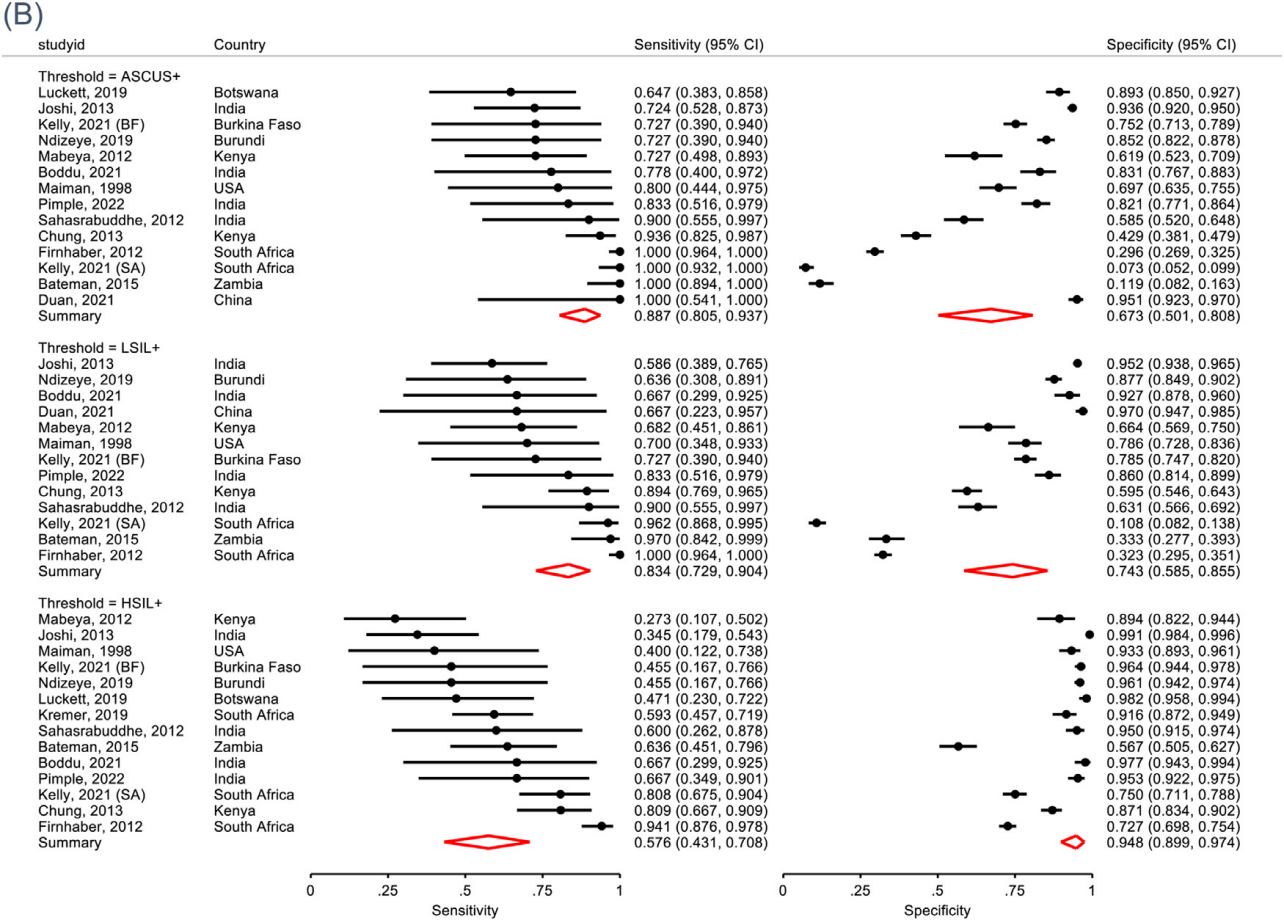
**Meta-analysis of diagnostic accuracy of cervical cytology for CIN2+ (A) and CIN3+ (B) among WLHIV in 20 studies, ranked according to sensitivity**. CIN2+= cervical intraepithelial neoplasia, grade 2; CIN3+= cervical intraepithelial neoplasia, grade 3; WLHIV=women living with HIV; ASCUS+= Atypical squamous cells of undetermined significance; LSIL+=low grade squamous intraepithelial lesions, or greater; HSIL+= high grade squamous intraepithelial lesions, or greater; Lao PDR=Lao People's Democratic Republic; Kelly, 2021 (BF) and Kelly, 202 (SA) refers to diagnostic accuracy estimates among women included in the Burkina Faso and South Africa sites, respectively; CI=confidence intervals.

Cytology ASCUS+ had pooled sensitivity of 85.1% (*I^2^=68%)* for CIN2+ (range: 57.5-100.0%) and 88.7% (*I^2^=52%*) for CIN3+ (range: 64.7-100.0). Specificity for <CIN2 was highly heterogeneous (range: 8.5-96.3%; Table 2; Figure 3). HSIL+ cytology had heterogeneous sensitivity for CIN2+ and CIN3+ (range CIN2+: 20.0% to 75.8%; CIN3+: 27.3% to 94.1%) and specificity (range: 58.3% to 94.1%; Figure 3) limiting the reliability of pooled estimates. In two studies^42^, ^45^ from settings with established cytology screening programme, HSIL+ had consistently higher sensitivity and specificity for CIN2+ (76.0%, 95%CI: 71.7-79.7 and 82.1%, 95%CI: 79.9-84.1, respectively; *data not shown*).

**Figure 4:**
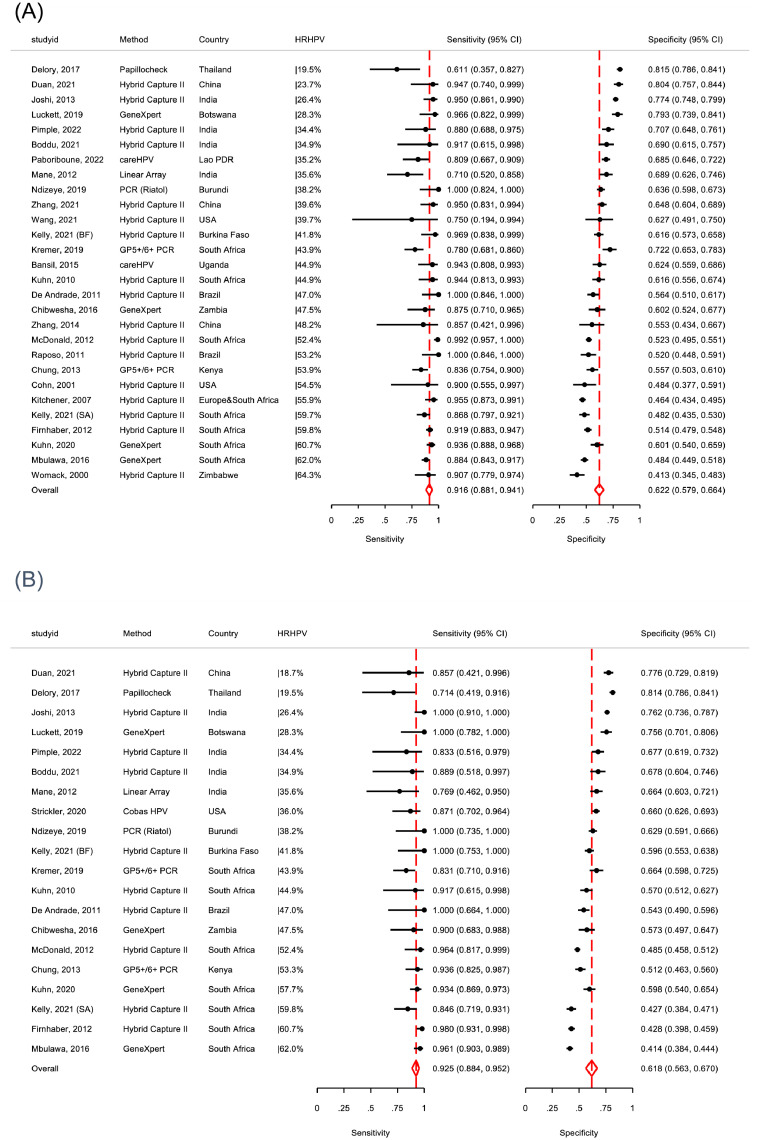
**Meta-analysis of diagnostic accuracy of HR-HPV DNA tests for CIN2+ (A) among WLHIV in 28 populations and CIN3+ (B) in 20 studies**. *HRHPV denotes proportion of women who tested positive for HR-HPV with respective test method. CIN2+= cervical intraepithelial neoplasia, grade 2; CIN3+= cervical intraepithelial neoplasia, grade 3; WLHIV=women living with HIV; HR-HPV=high risk human papillomavirus; Lao PDR=Lao People's Democratic Republic; Kelly, 2021 (BF) and Kelly, 202 (SA) refers to diagnostic accuracy estimates among women included in the Burkina Faso and South Africa sites, respectively; PCR=polymerase chain reaction.

In 28 studies, the pooled sensitivity of HR-HPV-DNA tests 91.6% (*I^2^*=45%) for CIN2+ and 92.5% (*I^2^*=32%) for CIN3+ (Tables 2, 3 and Figure 4). Specificity for <CIN2 was low for all test methods combined (62.2%, *I^2^=92%*) but increased with decreasing prevalence of HR-HPV among study participants (Figure 4). Four studies provided data to evaluate a restricted genotype approach targeting 8-HR types (HPV16/18/31/33/35/45/52/58) compared to up to 14-HR-HPV types (HPV16/18/31/33/35/39/45/51/52/56/58/59/66/68) in a head-to-head comparison.^39^, ^45^^,^^60^ The pooled sensitivity for CIN2+ of 8-HR restricted genotype approach was similar compared to that for HPV-DNA test (8-HR vs 14-HR: 86.4% vs. 91.5%, Relative Sensitivity=0.94, 95%CI: 0.88-1.00; Table 4, Appendix, page 16) but specificity was significantly higher (65.7% vs. 56.3%; Relative Specificity=1.18; 95%CI: 1.10-1.23). Similar findings were observed for CIN3+ detection (CIN3+: 88.3% vs. 92.5%; RSens=0.96, 95%CI:0.89-1.03). Modification to HR-HPV DNA test positivity thresholds (relative light unit [RLU] or PCR cycle thresholds) showed a 36% increase in specificity (RSpec=1.36, 95%CI: 1.27-1.47), though with 11% loss in sensitivity (RSens=0.89, 95%CI: 0.82-0.97; Appendix, page 17).

**Table 3:** Meta-analysis of diagnostic accuracy of cervical cancer screening strategies for CIN3+ among WLHIV.

	N studies	N WLHIV	CIN3+ prevalence, % (95%CI)	Test positive, % (95%CI)	Sensitivity for CIN3+ (%, 95%CI)	*I**^2^*	Specificity for CIN3+ (%, 95%CI)	*I**^2^*	*p-value*
**Screen approach**									
VIA – naked eye^a^									
Studies with ≥95% histology verification 31, 38, 39, 45, 47	5	1703	9.4 (5.8-12.9)	33.6 (22.7-44.5)	65.0 (52.9-75.4)	*41.6%*	71.5 (57.5-82.3)	*95.1%*	*0.38*
Studies with 50-95% histology verification 37, 42^,^^45^	3	1932	5.2 (0.8-9.6)	30.4 (16.6-44.2)	70.4 (61.4-78.0)	*1.0%*	72.2 (62.6-80.1)	*90.3%*	*0.11*
Studies with <50% histology verification 29, 36^,^^51^, ^53^	4	2386	3.1 (1.9-4.4)	20.0 (9.7-30.4)	83.0 (72.0-90.3)	*7.2%*	85.1 (70.3-93.2)	*96.8%*	*0.99*
***Cytology***									
Cytology ASCUS+ 25, 29, 32^,^^37^, ^39^, ^41^, ^42^, ^45^^,^^47^, ^48^^,^^51^, ^53^	14	6542	5.7 (4.0-7.3)	39.0(19.4-58.7	88.7 (80.5-93.7)	*51.8%*	67.3 (50.1-80.8)	*98.6%*	*0.22*
Cytology LSIL+ 25, 29, 37^,^^39^, ^41^, ^42^, ^45^, ^47^^,^^51^, ^53^	13	6005	5.6 (3.9-7.3)	34.6 (16.7-52.6)	83.4 (72.9-90.4)	*60.5%*	74.3 (58.5-85.5)	*98.3%*	*0.30*
Cytology HSIL+ 25, 29, 30^,^^31^, ^32^^,^^37^, ^39^, ^42^, ^45^^,^^47^, ^48^^,^^51^	14	6457	7.0 (5.0-8.9)	14.4 (9.0-19.8)	57.6 (43.1-70.8)	*72.1%*	94.8 (89.9-97.4)	*93.3%*	*0.70*
***HPV based tests***									
***All***^27^, ^28^, ^29^, ^30^, ^31^, ^32^, ^33^^,^^36^, ^37^, ^38^, ^39^^,^^,^^42^, ^45^^,^^49^^-^^51^^,^^60^^,^^61^	20	11649	6.2 (4.7-7.7)	42.2 (35.7-48.8)	92.5 (88.4-95.2)	*32.0%*	61.8 (56.3-67.0)	*95.6%*	*0.88*
Hybrid Capture II^b^^27^, ^29^, ^31^^,^^36^^,^^37^^,^^42^, ^45^^,^^50^	10	6276	4.2 (2.7-5.6)	42.7 (33.5-51.9)	95.3 (89.1-98.0)	*25.1%*	60.0 (51.9-67.6)	*96.1%*	*0.69*
GeneXpert^c^^32^, ^38^, ^49^^,^^60^	4	2036	14.9 (5.0-24.8)	56.3 (49.0-63.7)	94.4 (90.1-96.9)	*1.4%*	58.9 (46.0-70.7)	*94.7%*	*0.52*
GP5+/6+ 30, 39	2	738	13.3 (10.9-15.7)	49.6 (46.0-53.2)	87.9 (80.2-92.8)	*-*	56.8 (52.9-60.6)	*-*	*-*
***Restricted genotyping***									
8-HR [low threshold]^d^^39^, ^45^^,^^60^	4	2018	11.9 (3.9-19.8)	44.5 (34.8-54.1)	88.3 (80.0-93.5)	*33.9%*	62.6 (55.9-68.9)	*89.0%*	*0.84*
8-HR [high threshold]d ^45^^,^^60^	3	1651	12.3 (1.7-23.0)	33.9 (23.2-44.5)	81.9 (70.7-89.5)	*61.5%*	73.1 (67.0-78.4)	*83.9%*	*0.69*
OncoE6 (HPV16/18/45) 38, 51	2	879	2.2 (1.2-3.2)	3.4 (2.2-4.6)	46.9 (30.6-63.9)	*-*	98.0 (96.8-98.7)	*-*	*-*

*one study^45^ contributed diagnostic accuracy for two distinct populations in two countries; each country specific estimate is considered as a seperate study in the meta-analysis^;^ **p-value for publication bias; ^a^conducted by nurse/midwife; ^b^HC-II targets 13 HR types: HPV-16, -18, -31, -33, -35, -39, -45, -51, -52, -56, -58, -59, and -68; ^c^GeneXpert 5-channel targets 14 HR types: HPV-16, -18, -31, -33, -35, -39, -45, -51, -52, -56, -58, -59, 66 and -68; ^d^8 HR types: HPV 16; HPV 18 or 45; or HPV 31, 33, 35, 52, or 58; analysis includes two studies used HC-II and INNO-LiPA (positive for HC-II and any HPV16/18/45/31/33/35/52/58; low threshold=1RLU; high threshold=20RLU)^35^, ^45^ and one study used GeneXpert (low threshold=a higher number of replication cycles; high threshold=low number of replication cycles).^60^

**Table 4:** Pooled relative sensitivity and relative specificity of HR-HPV-DNA testing screening compared to VIA and cervical cytology for detection of CIN2+/CIN3+.

	CIN2+					CIN3+				
	N studies	Relative Sensitivity (95%CI)	p-value	Relative Specificity (95%CI)	p-value	N studies	Relative Sensitivity (95%CI)	p-value	Relative Specificity (95%CI)	p-value
**HR-HPV (14 HR types)**										
14-HR-HPV vs. VIA^24^^,^^29^, ^31^, ^37^, ^38^, ^39^, ^42^^,^^45^^,^^51^^,^	10	1.40 (1.27-1.56)	<0.001	0.77 (0.73-0.82)	<0.001	9	1.36 (1.22-1.53)	<0.001	0.74 (0.68-0.79)	<0.001
14-HR-HPV vs. Cytology ASCUS+^29^, ^32^^,^^37^^,^^39^, ^40^, ^42^^,^^45^^,^^46^^,^^51^	10	0.97 (0.93-1.01)	0.11	0.89 (0.85-0.94)	<0.001	7	1.06 (1.00-1.13)	0.07	0.96 (0.92-0.99)	0.02
14-HR-HPV vs. Cytology LSIL+^29^, ^37^, ^39^, ^42^^,^^45^^,^^46^^,^^51^^,^^,^^,^	8	1.00 (0.96-1.05)	0.87	0.83 (0.76-0.89)	<0.001	7	1.10 (1.01-1.19)	0.04	0.89 (0.84-0.95)	<0.001
14-HR-HPV vs. Cytology HSIL+^29^, ^32^, ^34^^,^^37^, ^39^, ^42^^,^^45^^,^^51^	9	1.55 (1.36-1.75)	<0.001	0.68 (0.61-0.76)	<0.001	8	1.38 (1.16-1.64)	<0.001	0.68 (0.59-0.78)	<0.001
14-HR-HPV alone vs. HPV ->VIA triage^32^^,^^39^, ^42^, ^45^^,^^51^^,^^a^	6	1.42 (1.22-1.66)	<0.001	0.68 (0.62-0.75)	<0.001	-	-	-	-	-
14-HR-HPV alone vs. HPV-> HSIL+ triage^39^, ^45^	3	1.37 (1.20-1.57)	<0.001	0.59 (0.50-0.70)	<0.001	-	-	-	-	-
**HR-HPV (8 HR types)**										
8-HR-HPV vs, 14 HR-HPV 39, 45^,^^60^	4	0.94 (0.88-1.00)	0.05	1.18 (1.10-1.23)	<0.001	4	0.96 (0.89-1.03)	0.21	1.19 (1.11-1.28)	<0.001
8-HR-HPV vs. VIA^39^, ^45^	3	1.44 (1.23-1.69)	<0.001	0.92 (0.86-0.99)	0.02	3	1.36 (1.13-1.64)	0.001	0.89 (0.83-0.96)	0.002
8-HR-HPV vs. Cytology HSIL+^39^, ^45^	3	1.23 (1.10-1.39)	<0.001	0.59 (0.44-0.79)	<0.001	3	1.11 (0.97-1.28)	0.14	0.62 (0.50-0.76)	<0.001
8-HR-HPV vs. HPV->VIA triage^39^, ^45^	3	1.59 (1.29-1.95)	<0.001	0.77 (0.71-0.83)	<0.001	-	-	-	-	-
8-HR-HPV vs. Cytology HSIL+^39^, ^45^	3	1.31 (1.14-1.51)	<0.001	0.56 (0.39-0.81)	0.002	-	-	-	-	-

a For comparison of stand-alone test in screening vs. triage strategy, all women who were screened were included as denominator for both screening and triage scenarios (i.e. for the triage group, women who were screen-negative were considered screen-triage negative).

In five studies,^29^, ^39^, ^42^, ^45^ co-testing using HPV-DNA test with either VIA or cytology had similar sensitivity for CIN2+ as HR-HPV-DNA test as single screening test (95.4% and 95.5%, respectively) but with lower specificity (48.6% and 56.8%, respectively) yielding higher proportion of screen-positive women than either test alone (Table 2).

Fifteen studies evaluated the diagnostic accuracy of HR-HPV-DNA tests in head-to-head comparisons with at least two other screening tests.^24^, ^29^, ^31^, ^32^, ^34^, ^37^, ^38^, ^39^, ^40^, ^42^^,^^45^^,^^46^^,^^51^ In ten studies,^24^, ^29^, ^31^, ^37^, ^38^, ^39^, ^42^^,^^45^^,^^51^) HR-HPV-DNA tests were more sensitive for CIN2+ and CIN3+ compared to VIA (CIN2+: pooled Relative Sensitivity[RSens]=1.40, 95%CI: 1.27-1.56; CIN3+: 1.36, 95%CI: 1.22-1.53) but less specific for <CIN2 (pooled Relative Specificity[RSpec]=0.77, 95%CI: 0.73-0.82; Table 4). HR-HPV-DNA tests were also more sensitive than cytology HSIL+ but less specific.

In six studies^32^^,^^39^, ^42^, ^45^^,^^51^ among HR-HPV-positive WLHIV, the sensitivity and specificity of VIA for CIN2+ (57.4%, 95%CI: 41.8-71.7, *I^2^*=58% and 79.9%, 95%CI:64.4-89.7, *I^2^*=91%, respectively) was similarly heterogeneous as it was for VIA alone (Appendix, page 18). In all studies, VIA operators were reported to be blinded to the HPV test result. Similar findings were observed for cervical cytology following an HPV-positive test in 5 studies^32^^,^^39^, ^42^, ^45^ (Table 2).

Compared to a scenario where women underwent screening with 14-HR-HPV-DNA test without triage in six studies,^32^^,^^39^, ^42^, ^45^^,^^51^ triage of those HPV-positive women using VIA was less sensitive for CIN2+ (VIA triage vs. HPV-DNA screen: 64.4% vs. 91.6%; RSens=0.70. 95%CI: 0.60-0.82) but more specific (88.1% vs. 60.0%; RSpec=1.47, 95%CI: 1.33-1.62). Similar findings were observed for HSIL+ triage of HPV-positive women (Table 4). 8HR-HPV-DNA as a stand-alone test in screening without triage had similar sensitivity to 14HR-HPV-DNA and specificity equivalent to VIA as a stand-alone in screening, though number of studies was small.

The main variation in study methodology for studies evaluating VIA and cytology was linked to endpoint ascertainment (Appendix, page 9-12). Sub-group meta-analyses were conducted according to the proportion of women with cervical biopsy and histology verification of disease, as previously described for VIA. Few studies^29^, ^43^, ^51^ documented frequent training, supervision and experience of VIA operators, and although sensitivity for CIN2+ was high in these studies (83.8%, 95%CI: 75.9-89.5; *I^2^*=6%), the proportion of women who underwent biopsy and histology verification was low (range: 8% to 23%). Cytology HSIL+ had the highest joint sensitivity and specificity in studies where the majority of women had histological verification of disease, and in settings with external quality assessment (EQA) programmes in place. In three studies with biopsy rate between 74% and 100%, two of which had established cytology programme in place,^39^, ^42^, ^45^ sensitivity of HSIL+ for CIN2+ was 74.5% (95%CI: 69.4-79.0, *I^2^=15.8%*) and specificity was 89.0% (95%CI: 75.8-95.5, *I^2^=92.2%) (data not shown*).

**Figure 5:**
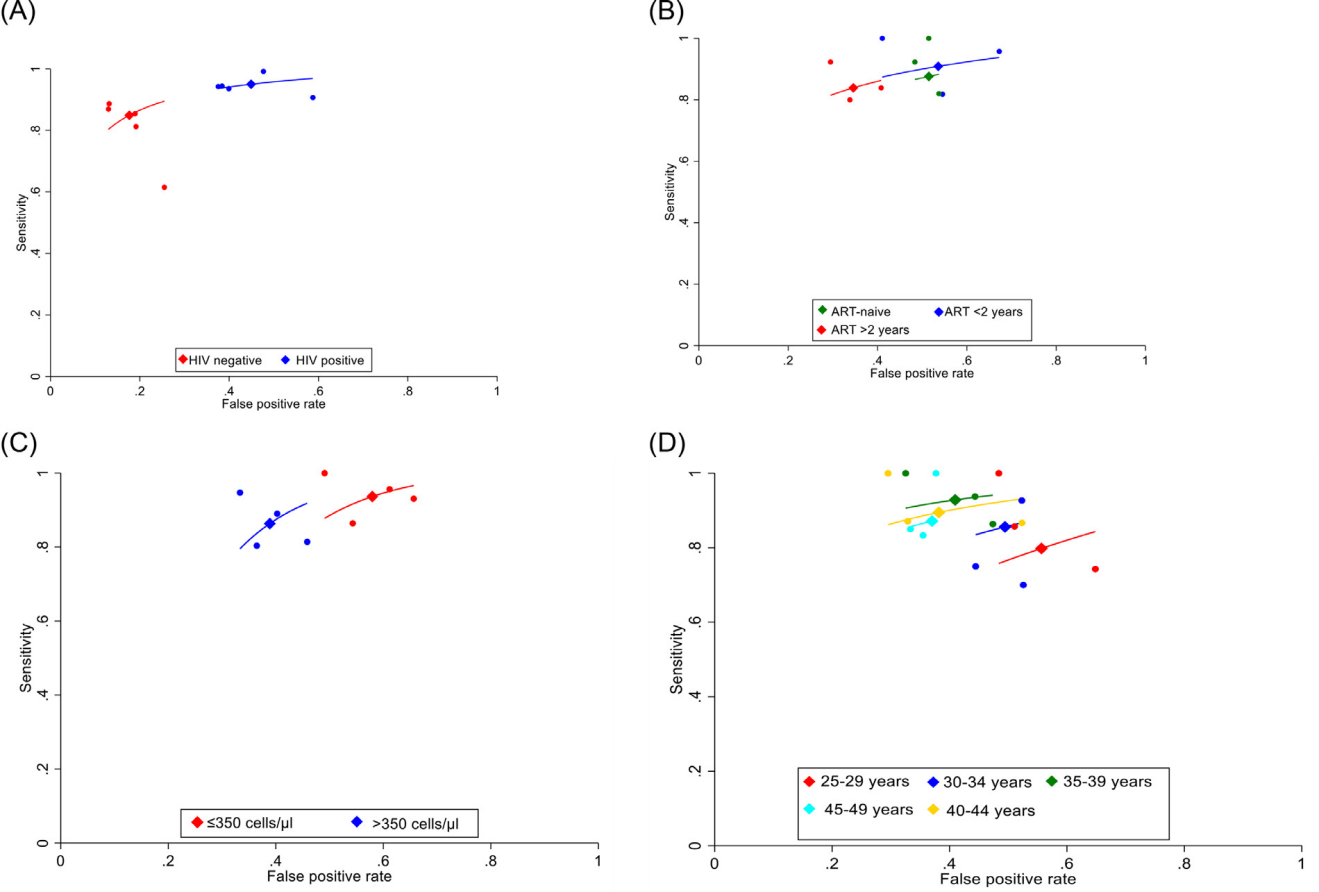
**Meta-analysis (SROC) of performance of HR-HPV-DNA testing for CIN2+ among WLHIV by HIV status in 5 studies (A), ART status in 3 studies (B), CD4+ count (cells/µl) in 4 studies (C) and by age in 3 studies (D)**. Tests evaluated include careHPV,^24^ Hybrid Capture-II^31^^,^^35^^,^^45^^,^^50^ and GeneXpert.^60^ Where multiple estimates given, estimates using cervical specimens^24^ and using standard threshold levels^35^^,^^60^ were selected. SROC= Summary Receiver Operating Characteristic; CIN2+= cervical intraepithelial neoplasia, grade 2; CIN3+= cervical intraepithelial neoplasia, grade 3; HIV=human immunodeficiency virus; WLHIV=women living with HIV; HR-HPV=human papillomavirus; DNA=Deoxyribonucleic acid; ART=antiretroviral therapy. Tests evaluated include careHPV,^27^ HC-II^39^^,^^41^^,^^51^^,^^52^ and GeneXpert.^25^ Where multiple estimates given, estimates using cervical specimens^27^ and using standard threshold levels^25^^,^^52^ were selected. SROC= Summary Receiver Operating Characteristic.

The accuracy of HPV-DNA test was influenced by participant selection, namely factors associated with immunosuppression (Appendix, page 13-14, 21-22). In five studies that allowed comparison of the diagnostic accuracy of HPV-DNA tests by HIV status,^24^, ^31^, ^35^, ^50^^,^^60^ the sensitivity of HPV-DNA was higher in WLHIV compared to women without HIV (95.0% vs. 84.9%; Relative Sensitivity=1.12, 95%CI: 1.05-1.19; Figure 5), but the specificity was lower (55.0% vs. 82.3%; Relative Specificity=0.67, 95%CI: 0.62-0.72). In two studies,^39^, ^45^ the specificity of HPV-DNA tests was higher in women on prolonged duration ART (≥2 years) compared to women on short-duration ART (<2 years; Relative Specificity= 1.42, 95%CI: 1.23-1.63) and ART-naïve women (Relative Specificity=1.35, 95%CI: 1.19-1.53) with no difference in sensitivity (Appendix, page 21). The sensitivity of HPV-DNA for CIN2+ was also similar in WLHIV with high compared to low CD4+ count (>500 vs. ≤500 cells/µl: 82.2% vs. 89.6%; Relative Sensitivity=0.92, 95%CI: 0.82-1.03) but with higher specificity (64.8% vs. 50.3%; Relative Specificity=1.29, 95%CI: 1.17-1.43 (Appendix, page 22).

In age-stratified analyses, sensitivity of VIA for CIN2+ was lower in older compared to younger women in two studies^39^, ^45^ (from 59.4% in ages 25-29 to 25.0% among women ≥50 years) but specificity did not vary (Appendix, page 19-20). By contrast, the specificity of HR-HPV-DNA tests increased with increasing age (specificity for <CIN2: 40.0%, in ages 20-24 to 70% among women ≥50 years; Figure 5)^39^, ^45^ but sensitivity did not vary. The test positivity decreased with increasing age, corresponding with lower HR-HPV prevalence in older age groups.

## Discussion

In this review of studies evaluating the performance of cervical cancer screening approaches for cervical precancer detection in WLHIV, we found that HR-HPV-DNA tests demonstrated consistently higher sensitivity and identified more cervical precancer cases than VIA or cervical cytology. However, approximately half of all WLHIV had a positive HR-HPV-DNA result, with greatest proportion in sub-Saharan Africa settings where HR-HPV prevalence is highest,^62^ resulting in low specificity of HPV-DNA tests. Optimal triage approaches for HR-HPV positive WLHIV remain uncertain. A restricted 8-HR-HPV genotype approach would overcome this due to its increased specificity, with minimal loss in sensitivity.

Of the estimated 19.3 million WLHIV in 2020,^63^ the majority live in low- and middle-income countries where cervical cancer incidence is highest^64^ but where cervical cancer screening coverage, linkage to care and HPV vaccination is low in the general population^3^^,^^4^ and largely unknown for WLHIV. Where screening is available, it is often opportunistic and VIA is the most common screening test used^4^ despite being less effective in preventing CIN2+ incidence in WLHIV compared to HPV-DNA test.^31^ Our review reports wide variability in sensitivity and specificity estimates for VIA as has been observed in the general population,^7^ resulting from the subjective nature of the exam. We were unable to assess the impact of quality assurance on VIA as no studies reported specifically on this. However, digital cervicography,^65^ peer feedback and review of charts,^66^ use of mobile health applications or telemedicine consultations that improve QA of nurse-led VIA^67^ have all previously been shown to improve accuracy to detect cervical precancer. The variability in VIA estimates may also be attributed to potential gold standard misclassification and partial verification bias. VIA and colposcopy are correlated as both rely on visual detection of lesions. Both the sensitivity and specificity of VIA may be overestimated if colposcopy directs biopsies for gold standard histological verification at the same areas that are detected with VIA.^7^^,^^68^ In our review, we conducted an analysis restricted to studies within which women were subjected to both directed biopsy of colposcopy abnormal areas and random biopsy of colposcopy normal area and endocervical curettage, ensuring the majority of women had an histological endpoint. In these studies, VIA had lower sensitivity and specificity for CIN2+ detection when compared to studies where biopsy was indicated for women with abnormal colposcopy only. Furthermore, VIA and colposcopy may miss lesions in the endocervix, in particular in older postmenopausal women as the squamo-columnar junction recedes into the endocervical cancer. In WLHIV, the co-existence of other sexually transmitted infections and associated inflammation also represent challenges compromising visualisation and interpretation.^69^ AVE incorporating digital imaging technology and use of artificial intelligence for interpretation or sending images to specialist centres could improve accuracy and reproducibility of visual inspection methods and could be used for quality control. AVE applied to cervigrams has been evaluated in women without HIV and has shown higher accuracy compared to conventional cytology^70^ but have not yet been studied in WLHIV, although studies are ongoing.

Although HR-HPV-DNA tests had the highest sensitivity of all screening approaches to detect CIN2+/CIN3+, its low specificity presents a challenge in a high HPV prevalence setting. An analysis evaluating the relationship between HR-HPV prevalence and the specificity of HR-HPV DNA testing to rule out CIN2+ reported that for a 10% increase in HR-HPV prevalence, HPV-DNA test specificity decreased by 8%.^9^ The high prevalence of HPV among WLHIV, often with multiple types, many of which may be transient infections^11^ results in low specificity of HPV-DNA tests. In a small number of studies, HPV-DNA tests had higher specificity among women with high CD4+ cell count and/or prolonged ART use, corresponding with lower HR-HPV prevalence with no change in sensitivity.^71^ In the universal ART era, when women are treated before profound immunosuppression,^72^ specificity of HR-HPV DNA tests may increase to become similar to that observed in women without HIV. Higher specificity can be achieved with HPV-DNA tests targeting a restricted set of genotypes (HPV16/18/45/31/33/35/52/58) with minimal impact on sensitivity. This single screening approach had better joint sensitivity and specificity compared to triage (using VIA or cytology) of HPV-positive women and may be less complex and costly to perform. Additional modifications including increasing test cut-off used to define screen-test positivity further increased specificity.^16^ Any associated loss in sensitivity with this approach may need to be balanced with capacity to refer HR-HPV positive women for colposcopy and treatment. Further evidence on the long term risk of cervical precancer in HPV negative WLHIV is also needed to inform on optimal interval of screening. HPV-DNA based tests provide the added advantage of allowing for self-sampling which may facilitate screening participation and be a cost-effective approach.^73^ Molecular methods that could distinguish persistent from transient infection, including tests targeting DNA methylation, E6/E7 oncoproteins and mRNA^74^ may be useful in WLHIV and warrant further evaluation of accuracy and feasibility in resource-constrained settings. Artificial intelligence based methods could also assist triage of HR-HPV-positive women in the absence of other molecular markers.^75^

Cervical cytology had variable sensitivity and specificity as observed for VIA, possibly resulting from differences in operator experience, training and implementation of EQA. In settings with cytology-based screening programmes incorporating EQA, HSIL+ had the best combination of sensitivity and specificity and may be an option for triage of HR-HPV positive WLHIV.

This is the first review to estimate the diagnostic accuracy of commonly available cervical cancer screening strategies for WLHIV and has investigated the sources of heterogeneity in performance, in particular the association of HIV related factors on HPV-DNA test specificity. This review has several limitations. Due to the very large methodological and statistical heterogeneity observed in the studies we reviewed, we cannot consider the pooled accuracy estimates of VIA or cytology as reliable. We did however attempt to adjust for differences in gold standard verification approaches in stratified analysis. Further, the relative accuracy estimates comparing screening tests are less likely to be influenced by these methodological differences as only those studies that conducted direct head-to-head comparison of tests were included. We were also unable to assess the impact of quality of training and QA on the diagnostic accuracy of VIA as this was not consistently reported across studies. Few (20%) studies reported on a priori sample size calculations to estimate precision and power to achieve study objectives, an essential item for reporting of diagnostic accuracy studies according to the 2015 Standards for Reporting Diagnostic Accuracy (STARD) statement,^76^ although they may be given in the individual unpublished study protocols. The majority of studies enrolled WLHIV prior to the availability of universal ART when guidelines recommended initiation of ART at lower CD4+ count (<500 cells/µl or <350 cells/µl), which may increase the likelihood of HR-HPV persistence and cervical lesion development.^77^ The lack of widespread evidence on cervical cancer screening among a contemporary cohort of WLHIV who may have started ART soon after HIV diagnosis and before profound immune suppression limits our understanding on performance of HPV-DNA tests in a population of WLHIV with well controlled HIV. Future studies among these women are needed.

Of the current screening strategies available, high performance tests such as HR-HPV-DNA tests demonstrated consistently higher sensitivity than VIA or cytology among WLHIV. Despite the lower specificity of HPV-DNA based methods targeting up to 14 HR types, limited evidence suggests that a restricted genotype approach may increase specificity, resulting in fewer referrals and clinic visits thereby reducing inconvenience to women and costs to the service. With earlier ART initiation and HIV virological control in the universal ART era, WLHIV may maintain a functionally complete mucosal immune response leading to a more rapid clearance of HR-HPV. Consequently, the diagnostic accuracy of HPV-DNA based tests may have improved specificity as observed in the general population. Optimal triage options for HPV-positive WLHIV remain unclear. More evidence is needed on the use of molecular diagnostic tools alone or in combination with visual methods or artificial intelligence among WLHIV, particularly in resource-constrained settings. Greater efforts are needed to make current diagnostic technologies more accessible and affordable in settings where the risk of cervical cancer and its associated morbidity and mortality are greatest,^78^ alongside the implementation of population-based screening approaches to increase screening coverage for the prevention of cervical cancer.^79^

## Contributors

HK, NB, SD, MA and SdS conceptualised the study, and developed the research protocol; HK and IJ identified articles for full-text review; HK and IJ and MA extracted data from studies that matched inclusion criteria; HK, IJ and MA had access to and verified the data; MC, SG, HS, XX and PM provided raw data for additional analyses; HK and MA did the statistical analyses with input from MC, NB and SD; HK, IJ, MC, PaM, SG, HS, XX, MS, NB, PM, SD, MA and SdS contributed to the writing of the manuscript; HK was responsible for decision to submit for publication.

## Data sharing statement

The dataset is available at Mendeley online repository at DOI: 10.17632/cn53hzsh5p.2.

## Declaration of interests

SdS is a member of the Data and Safety Monitoring Board of a screening trial in Zambia led by IARC evaluating cervical precancer recurrence after treatment and is on the Advisory Board of the HPV Prevention and Control Board. All other authors declare no competing interests.

## Appendix Supplementary materials

Image, application 1
