# Supplementary material for: Diagnostic accuracy of cervical cancer screening strategies for high-grade cervical intraepithelial neoplasia (CIN2+/CIN3+) among women living with HIV: A systematic review and meta-analysis
Source: eClinicalMedicine. 2022 Sep 27;53:101645. doi: 10.1016/j.eclinm.2022.101645 (PMC9520209; doi:10.1016/j.eclinm.2022.101645)
Supplement: Supplementary file 1 [file mmc1.docx]

**Diagnostic accuracy of cervical cancer screening strategies for high-grade cervical intraepithelial neoplasia (CIN2+/CIN3+) among women living with HIV: a systematic review and meta-analysis**

**Supplementary information**

Contents

[Search strategy 2](#_Toc110619491)

[Quality Assessment of Diagnostic test Accuracy Studies (QUADAS) items 3](#_Toc110619492)

[Supplementary Table 1. Characteristics of 38 included studies, in alphabetical order 6](#_Toc110619493)

[Methodological quality of studies evaluating diagnostic accuracy of VIA for CIN2+/CIN3+ 9](#_Toc110619494)

[Methodological quality of studies evaluating diagnostic accuracy of cervical cytology for CIN2+/CIN3+ 11](#_Toc110619495)

[Methodological quality of studies evaluating diagnostic accuracy of HPV-DNA tests for CIN2+/CIN3+ 13](#_Toc110619496)

[Supplementary Table 2. Meta-analysis of diagnostic accuracy of VIA-DC and VILI for CIN2+ and CIN3+ among WLHIV 16](#_Toc110619497)

[Supplementary Figure 4. Meta-analysis of performance of HPV tests for CIN2+ (top panel) and CIN3+ (bottom panel) among WLHIV by restricting targeted genotype approach and threshold in 4 studies 17](#_Toc110619498)

[Supplementary Figure 5. Meta-analysis of diagnostic accuracy of triage of HR-HPV positive women for CIN2+ (A) and CIN3+ (B) among WLHIV in 6 studies 19](#_Toc110619499)

[Supplementary Table 3. Diagnostic accuracy of screening tests (HPV-DNA, VIA and cytology) by age group in three studies 20](#_Toc110619500)

[Supplementary Table 4. Pooled relative sensitivity and relative specificity of HPV testing screening compared to VIA and cervical cytology for detection of CIN2+, stratified by age 21](#_Toc110619501)

[Supplementary Table 5. Diagnostic accuracy of screening tests (HPV-DNA, VIA and cytology) by ART status group in three studies 22](#_Toc110619502)

[Supplementary Figure 6. Meta-analysis of diagnostic accuracy of HPV-DNA tests for CIN2+ (panel A and B) and CIN3+ (panel C and D) by CD4+ count in five studies 23](#_Toc110619503)

[PRISMA 2020 Checklist 25](#_Toc110619504)

[References 28](#_Toc110619505)

# Search strategy

Search strategy in MEDLINE, EMBASE, Global Health and Wiley Cochrane libraries on 21-22 April 2020 and updated on 28 January 2021, 13 August 2021 and 15 July 2022. Terms adapted for each database. Full search strategy and results available upon request. No language limits were utilized.

Cervix.mp. or Cervix Uteri/ [MeSH terms] OR cervical [Title/Abstract] OR cervico [Title/Abstract]

AND

cancer [Title/Abstract] OR cancerous [Title/Abstract] OR precancer* [Title/Abstract] OR pre-cancer* [Title/Abstract] OR premalignant [Title/Abstract] OR carcinoma* [Title/Abstract] OR adenocarcinoma* [Title/Abstract] OR neoplas* [Title/Abstract] OR dysplas* [Title/Abstract] OR dyskaryoisis [Title/Abstract] OR squamous [Title/Abstract] OR CIN* [Title/Abstract] OR CINI* [Title/Abstract] OR CIN2* [Title/Abstract] OR CINII* [Title/Abstract] OR CIN3* [Title/Abstract] OR CINIII* [Title/Abstract] OR cervical intraepithelial neoplasia [MeSH Terms]

AND

visual* inspect* [Title/Abstract] OR visual screening [Title/Abstract] OR visual* examin* [Title/Abstract] OR visual* evaluat* [Title/Abstract] OR visual* detect* [Title/Abstract] OR visual test [Title/Abstract] OR visual* tested [Title/Abstract] OR visual* testing [Title/Abstract] OR visual tests [Title/Abstract] OR naked eye [Title/Abstract] OR acetic acid [MeSH terms] OR acetowhite [Title/Abstract] OR aceto-white [Title/Abstract] OR Iodine [MeSH terms] or Coloring Agents [MeSH terms] OR magnifying [Title/Abstract] OR magnified [Title/Abstract] OR magnification [Title/Abstract] OR aviscope [Title/Abstract] OR downstaging [Title/Abstract] OR automated visual examination [Title/Abstract] OR AVE [Title/Abstract] OR pap [Title/Abstract] OR Papanicolaou Test [MeSH terms] or Papanicolaou [Title/Abstract] OR vagina [Title/Abstract] OR cervical [Title/Abstract] OR cervix uteri [MeSH terms] or cervico OR smear* [Title/Abstract] OR test [Title/Abstract] OR tests [Title/Abstract] OR testing [Title/Abstract] OR tested [Title/Abstract] OR swab* [Title/Abstract] OR scrap* [Title/Abstract] OR Cytodiagnosis [MESH] OR Cell Transformaton, Viral [MeSH terms] OR Cytopathogenic effect, viral [MeSH terms] OR SIL [Title/Abstract] OR HSIL [Title/Abstract] OR H-SIL [Title/Abstract] OR LSIL [Title/Abstract] OR L-SIL [Title/Abstract] OR ASCUS [Title/Abstract] OR Atypical squamous cells of the cervix [MeSH terms] OR ASC-US [Title/Abstract] OR AS CUS [Title/Abstract] OR Papillomaviridae [MeSH terms] OR Alphapapillomavirus [MeSH terms] OR DNA, viral [MeSH terms] OR Papillomavirus Infections [Title/Abstract] OR Papillomavirus Infections [MeSH terms] OR Tumor Virus Infections [MeSH terms] OR cervix uteri [MeSH terms] OR HPV [Title/Abstract] OR human papillomavirus [Title/Abstract] OR papillomaviridae [Title/Abstract] OR PCR [Title/Abstract] OR Polymerase Chain Reaction [MeSH terms] OR hybrid capture [Title/Abstract] OR HC2 [Title/Abstract] OR HCII [Title/Abstract] OR HC 2 [Title/Abstract] OR HC II [Title/Abstract] OR DNA, viral [MeSH terms] OR CareHPV [Title/Abstract] OR GeneXpert [Title/Abstract] OR Xpert [Title/Abstract] OR OncoE6 [Title/Abstract] OR genotyping [Title/Abstract]

AND

HIV infections [MeSH terms] OR HIV [MeSH terms] OR hiv.ti,ab hiv1.ti,ab [Title/Abstract] OR hiv2.ti,ab [Title/Abstract] OR human immunodeficiency virus.ti,ab. [Title/Abstract] OR human immunedeficiency virus.ti,ab. [Title/Abstract] OR human immuno-deficiency virus.ti,ab. [Title/Abstract] OR human immune-deficiency virus.ti,ab. [Title/Abstract] OR (human immun* adj3 deficiency virus).ti,ab. [Title/Abstract] OR acquired immunodeficiency syndrome.ti,ab. [Title/Abstract] OR acquired immunedeficiency syndrome.ti,ab. [Title/Abstract] OR acquired immuno-deficiency syndrome.ti,ab. [Title/Abstract] OR acquired immune-deficiency syndrome.ti,ab. [Title/Abstract] OR (acquired immun* adj3 deficiency syndrome).ti,ab. [Title/Abstract]

# Quality Assessment of Diagnostic test Accuracy Studies (QUADAS) items

Adapted from ^(1)^

*Participant selection*

- ***Item 1*** of the QUADAS tool (representative spectrum of participants) will be scored as 'yes' if the tests are done on a representative population of WLHIV attending cervical cancer screening not being followed up for cervical abnormalities (i.e. not enriched for CIN2+), and ‘no’ if enriched for CIN2+ by inclusion of women referred to colposcopy clinics.
- Given that the history of immunosuppression (as measured by nadir CD4+ count) is a predictor of HR-HPV persistence, CIN2+ incidence and progression and cervical cancer, studies will be assessed according to period of recruitment of participants in the study (pre-combination ART era, early cART era when ART was initiated at <350 cells/µl, and universal ART era when ART was initiated irrespective of CD4+ count). Studies will be scored as ‘yes’ if participants are more representative of contemporary cohort of WLHIV (i.e. majority taking ART, majority of ART users with suppressed HIV-1 viral load, high median CD4+ count [>500 cells/µl]; representing future cohorts of WLHIV who may control HIV disease). The item will be scored as 'no' if the majority of the population are representative of historical cohort of WLHIV (i.e. smaller proportion taking ART, low median CD4+ count [≤500 cells/µl]) and 'unclear' if there is not sufficient information.
- In addition, for studies evaluating VIA, the items will be scored ‘no’ if women with symptoms/diagnosis of STI were excluded if they represent a large proportion of women enrolled (as this may impact VIA observation and WLHIV are at high risk of STI, cervicitis and associated inflammation).
- Score ‘no’ if low proportion taking ART, or women with STI excluded
- Item 2 of the QUADAS tool (selection criteria clearly described) will not be used as suggested in Chapter 9 of the *Cochrane Handbook for Diagnostic Test Accuracy Reviews^(2)^*.

*Reference test*

- **Item 3** (acceptable reference standard) **will not be used** as suggested in Chapter 9 of the *Cochrane Handbook for Diagnostic Test Accuracy Reviews^(2)^.* Eligible studies had to have histology verified endpoint as reference standard. Studies using cytology-verification only were excluded from this review.
- **Item 4** of the QUADAS tool (acceptable delay between tests) will be scored as 'yes' if the total interval between VIA, human papillomavirus (HPV) testing, cytology and verification with the reference standard was less than 12 weeks, as the status of the condition is unlikely to change within this time period. The item will be scored 'no' if the interval was equal to or more than 12 weeks, and 'unclear' if there is not sufficient information.
- **Item 5** of the QUADAS tool (partial verification avoided) will be scored as 'yes' if all women tested with index test (VIA, cytology or HPV testing) were referred to colposcopy with directed biopsy (or better including random) or when all women being positive for at least one screen test were referred together with a random sample of women being negative for all screen tests. The item will be scored 'no' if the selection of women who were referred to colposcopy-biopsy is influenced by the results of the screening tests (i.e. if all screen-positives are verified and not all screen-negatives). Women who were HPV negative are considered to be low risk of disease and colposcopy-histological verification would not be considered necessary. The item will be scored as 'unclear' if there is not sufficient information.
- **Item 6** of the QUADAS tool (differential verification avoided) will be scored as 'yes' if all women who had disease status verification, had this done by the same method (i.e. all women underwent colposcopy with directed biopsies as minimum, or better yet with random biopsy of normal looking quadrants (so that 100% of women have histological verification), or when proportion of women with biopsy and histological verification is greater than 70%). The item will be scored 'no' if the method of verification differed between groups of participants (i.e. histological verification among colposcopy abnormal women and no histological verification of colposcopy normal or if only screen-positive women were referred to colposcopy followed by directed biopsy (with or without random biopsy), and 'unclear' if there is not sufficient information.
- **Item 6x (misclassification bias avoided)** will be scored ‘yes’ if all CIN2+ histologic specimens and a random sample of negative biopsies were independently reviewed by a, or several, external pathologist(s), or diagnosis was reached by consensus of more than one pathologist. The item will be scored ‘no’ if there was no independent review of pathology, and ‘unclear’ if there is not sufficient information.
- **Item 7** of the QUADAS tool (incorporation avoided; i.e. if the reference standard used for disease status verification is not composed in any part by the index test) is not applied as only studies reporting some histological verification were included.

*Index test*

- For VIA**, Item 8** of the QUADAS tool (sufficient index test description) was composed of three parts. Item 8A will be scored as ‘yes’ if operator (nurse or colposcopist) and their level of training was described and will be scored 'no' if no description of operator. Item 8B will be scored ‘yes’ if the operator conducting VIA is independent of the operator conducting colposcopy (VIA and colposcopy blinded), and ‘no’ if the operator conducting VIA and colposcopy is the same person, or VIA and colposcopy conducted by different persons but at the same time (whereby operator conducting VIA could be influenced by colposcopist). Item 8C will be scored as ‘yes’ if quality control of reading is implemented (using digital cervicography post-exam and supervision with clinical staff; note does not change the original VIA diagnosis) and ‘no’ If there is no QC. For all 8A to 8C, the item will be scored 'unclear' if there is not sufficient information.
- For HPV test, Item 8 was composed of two parts. Item 8A will be scored as ‘yes’ if HPV testing was well described in terms of where/who conducted the testing and ‘no’ if not described or unclear. Item 8B will be scored ‘yes’ if sample used for HPV testing was clinician collected cervical specimen (to rule out potential loss in sensitivity associated with self-collected vaginal specimens) and/or the appropriate sample collection medium (manufacturer approved) was used, and ‘no’ if sample other than cervical was used and/or sample medium other than manufacturer approved was used.
- **Item 9** of the QUADAS tool (sufficient reference standard description) will not be used as suggested in Chapter 9 of the *Cochrane Handbook for Diagnostic Test Accuracy Reviews^(2)^*
- **Item 10** of the QUADAS tool (index test results blinded) will be scored as 'yes' if the VIA operators, cytologists and the technicians interpreting the Pap smear and the HPV test were not aware of the colposcopy/biopsy results. The item will be scored 'no' if they were made aware of the reference standard results prior to the interpretation of the screening tests, and 'unclear' if there is not sufficient information given in the text.
- **Item 11** of the QUADAS tool (reference standard results blinded) will be scored as 'yes' if the colposcopists and the pathologists were not aware of the VIA, cytology and HPV test results when interpreting the results of the reference standard. The item will be scored 'no' if either was aware of the screening test results (which is the case in clinical practice), and 'unclear' if there is not sufficient information in the text.
- **Item 12** of the QUADAS tool (relevant clinical information) will be scored as 'yes' if the cytologist was aware of the woman's basic history (age, symptoms, previous cervical surgery). If the cytologist was not aware the item will be scored 'no', and if this information is not given in the text it will be scored 'unclear'. Item 12 applied only when cytology included in analysis (absolute and relative accuracy).
- **Item 13** of the QUADAS tool (un-interpretable results reported) will be scored as 'yes' if the numbers of inadequate VIA, cytology and HPV test results are given. It will be scored 'no' if the numbers of inadequate tests are not given, and 'unclear' if it is not certain whether all test results have been reported.

*Participant flow*

- **Item 14** of the QUADAS tool (withdrawals explained) will be scored as 'yes' if it is clear what happened to all participants who entered the study, including the withdrawals. The item will be scored 'no' if it is not explained why no outcome could be obtained for some women, and if it is not clear whether all participants who entered the study were accounted for it will be scored 'unclear'. Within this item, a sample size calculation was also considered

# Supplementary Table 1. Characteristics of 38 included studies, in alphabetical order

| **Author, year** | **City, Country** | **Recruitment period** | **Test strategies evaluated** | **N WLHIV** | **N HIV negative women** | **Recruitment population** | **Study design** | **Age (range)** | **Age, median or mean years (IQR)** | **ART users (%)** | **CD4+ count at screening, median (IQR)** | **HR-HPV prevalence**  **% (95%CI)** | **CIN2+ prevalence**  **% (95%CI)** |
| --- | --- | --- | --- | --- | --- | --- | --- | --- | --- | --- | --- | --- | --- |
| Bansil, 2015^(3)^ | Uganda | 2011 | VIA, CareHPV | 272 | 946 |  | Cross sectional | 25-60 | NR | NR | NR | 44.9 (38.8-51.0) | 12.9 (9.1-17.4) |
| Bateman, 2015^(4)^ | Lusaka, Zambia | 2008-2011 | VIA/DC, Cytology ASCUS+, LSIL+, HSIL+ | 303 | - | Matero public health clinic | Cross sectional | 20-45 | 32 (28–37) | 86.4% | 56.5% with baseline CD4+ <200 cells | N/A | 20.1 (15.8-25.1) |
| Boddu, 2021^(5)^ | New Delhi, India | 2014-2015 | VIA, cytology (ASCUS+), HC-II | 186 | 202 | WLHIV attending ART clinic, HIV negative women from urban community | Cross sectional | 30-59 | 36 | 72.8% | 305 (range: 39-1493) | 37.6% (WLHIV)  5.9% (HIV-negative) | 6.4% (WLHIV)  0.5% (HIV-negative) |
| Branca, 2001^(6)^ | Italy | NR, pre-2000 | Cytology ASCUS+, LSIL+ | 37 | 21 | Enrolled in the DIANAIDS study | Cross sectional | 17-45 | NR | NR | NR | N/A | 21.6 (9.8-38.2) |
| Chibwesha, 2016^(7)^ | Lusaka, Zambia | 2015 | DC, VIA, GeneXpert, OncoE6 | 198 | - | Cervical cancer screening integrated in PEPFAR-supported HIV care and treatment infrastructure. | Cross sectional | 18+ | 42 (34-47). | 90.0% | 456 (IQR: 328-590) | 47.5 (40.4-54.7) | 16.2 (11.3-22.0) |
| Chung, 2013^(8)^ | Nairobi, Kenya | 2009 | VIA, Cytology ASCUS+, LSIL+, HSIL+, HPV (GP5+/6+) | 453 | - | Women attending PEPFAR-supported HIV care and treatment | Cross sectional | 18-55 | 38 (35–43) | 75.0% | 371 [245–533] | 53.9 (49.1-58.5) | 24.3 (20.4-28.5) |
| Cohn, 2001^(9)^ | USA | 1994-1998 | Cytology (ASCUS, LSIL, HSIL), VIA, HC-II | 101 | - | WLHIV receiving primary care | Cross sectional | 18+ | 33 (20-56) | 0.0% | 271 (range, 6-562) | 54.5 (44.2-64.4) | 9.9 (4.9-17.5) |
| DeAndrade, 2011^(10)^ | Rio de Janeiro, Brazil | 1996-2007 | HC-II | 366 | - | Women enrolled in prospective open cohort at FIOCRUZ | Prospective | - | 34 (28–41) | 27.0% | 347 (193–546) | 47.0 (41.8-52.3) | 6.0 (3.8-9.0) |
| Delory, 2017^(11)^ | Thailand – 24 hospital sites across country | 2012-2013 | Papillocheck HPV genotyping | 816 | - | WLHIV receiving ART enrolled in existing cohort | Cross-sectional | - | 40 (37-45) | 100% | 536 (412-670) | 22.1% | 2.2% |
| DeVuyst, 2015^(12)^ ^a^ | Nairobi, Kenya | 2009 | HPV+ 🡺 HPV16/18, Cytology ASCUS/LSIL/HSIL, VIA, human DNA methylation (*CADM1/MAL/MIR124-2*) | 248 | - | HR-HPV+ WLHIV enrolled in cervical cancer screening study^(8)^ | Cross sectional | 18-55 | 37 | 72.0% | 331 | 100.0% | 37.5 (31.5-43.8) |
| Duan, 2021^(13)^ | Yunnan, China | 2019-2020 | VIA/VILI, HC-II, Cobas, Sansure, Cytology (LBC) | 372 | - | WLHIV on ART attending routine HIV care | Cross sectional | 18+ | 40 (35–47) | 100.0% | 550 (401–704) | 31.7 | 5.1 |
| Firnhaber, 2012^(14)^ | Johannesburg, South Africa | 2009-2011 | VIA (nurse and DC), Cytology (ASCUS, LSIL, HSIL), HC-II - co-test and sequential | 1193 | - | WLHIV attending routine HIV care | Cross sectional | 18-65 | 38 (32-43) | 93.1% | 394 (253-572) | 59.8 (57.0-62.6) | 26.0 (23.5-28.6) |
| Huchko 2015^(15)^ | Kisumu, Kenya | 2007-2010 | VIA, VIA/VILI | 324 | - | WLHIV attending cervical cancer screening integrated in HIV care | Cross sectional | 23-65 | 35 | 35% (VIA/VILI) and 59% (VIA) | approx. 30% <200 cells/mm3 | N/A | 12.5 (10.8-14.3) |
| Huchko, 2014^(16)^ | Kisumu, Kenya | 2011-2012 | VIA | 1432 | - | WLHIV attending cervical cancer screening integrated in HIV care | RCT | 23+ | 35 | 76.5% | 544 cells/µl (±257) | N/A |  |
| Joshi 2013^(17)^ | Maharashtra state, India | 2010-2011 | VIA/VILI, Cytology ASCUS+ ; LSIL+ ; HSIL+, HC-II | 1128 | - | WLHIV recruited for cervical cancer screening | Cross sectional | 21-60 | 60% 30-39yrs | 75.0% | 64% <200 at ART initiation | 26.4 (23.9-29.1) | 5.3 (4.1-6.8) |
| Kelly, 2021^(18)^ | Ouagadougou, Burkina Faso & Johannesburg, South Africa | 2011-2012 | VIA, VIA/VILI, Cytology (ASCUS, LSIL, HSIL), HC-II, careHPV | 554 (BF); 576 (SA) | - | WLHIV attending routine HIV care | Prospective | 25-50 | 34 (30-40) | 67.0% (BF); 64.0% (SA) | BF: 446 (IQR, 309-600)  SA: 420 (IQR, 279-567 | BF: 41.8 (37.6-46.0)  SA: 59.7 (55.5-63.7) | BF: 5.8 (4.0-8.1)  SA: 22.5 (19.2-26.2) |
| Kitchener, 2007^(19)^ | Dublin, Edinburgh, London, Milan, Paris, Warsaw, Cape Town | 2000-2004 | Cytology ASCUS+, LSIL+, HC-II | 1534 | - | WLHIV attending routine HIV care | Cross sectional | 29-37 | 33 | 0% in SA; approx. 46% Europe (?) |  | 55.9 (53.4-58.4) | 9.5 (8.0-11.0) |
| Kremer, 2019^(20)^ | Pretoria, South Africa | 2013-2015 | Cytology HSIL+, HPV (GP5+/6+), human DNA methylation (*FAM19A4/miR124-2*) | 285 | - | WLHIV attending gynaecological outpatient clinic | Cross-sectional | 18+ | 40 (35-46) | 99% | 514 (380–720) | 43.9 | 20.7 (16.1-25.9) |
| Kuhn, 2020^(21)b^ | Cape Town, South Africa | 2015-2016 | GeneXpert | 446 | 482 | Women recruited from primary care (64%) and referral colposcopy clinic (36%) | Cross sectional | 30-65 | Median range: 38-44 | 80-88% | 455 (IQR 300–630). | 48.3% (Screening)  90.1% (Colposcopy)  Overall: 60.7 (56.0-65.2) | 15.3% (screening)  55.4% (colposcopy)  Overall: 38.7 (34.1-43.4) |
| Kuhn, 2010^(22)^ | Cape Town, South Africa | 2000-2002 | VIA, HC-II | 312 | 1841 | Unscreened women attending ambulatory women’s health clinics | RCT | 35-65 | 40.5 | NR (0%?) | NR | 44.9 (39.3-50.6) | 11.5 (8.2-15.6) |
| Luckett, 2019^(23)^ | Gaborone, Botswana | 2018 | GeneXpert HR-HPV+ 🡺 VIA, Cytology ASCUS+, HSIL+; HPV+ -> colpo | 290 | - | WLHIV attending infectious disease care clinic | Prospective | 25+ | 46 (42–52) | 100.0% | 71% >500 | 28.3 (23.2-33.8) | 10.0 (6.8-14.0) |
| Mabeya, 2012^(24)^ | Eldoret, Kenya |  | VIA, cytology | 150 | - | WLHIV attending routine HIV care | Cross sectional | 15-49 | 34 (20-45) | 67.1% | 438 (10-1,198) | N/A | 30.7 (23.4-38.7) |
| Maiman, 1998^(25)^ | Brooklyn, USA | 1990-1993 | Cytology | 248 | - | WLHIV attending routine HIV care | Cross sectional | - | 34 | NR | NR | N/A | 11.7 (8.0-16.4) |
| Mane, 2012^(26)^ | Pune, India | NR | HPV genotyping (Linear Array) | 275 | - | Women attending outpatient gynecology clinic | Cross sectional | - | 32 (±5) | 55.6% | 372 (241–556) | 35.3% | 11.3% |
| Mbulawa, 2016^(27)^ | Johannesburg, South Africa | 2009-2011 | GeneXpert | 1161 | - | WLHIV attending routine HIV care, as for ^(14)^ | Cross sectional | 18-65 | 38 (32-43) | 93.1% | 394 (253-572) | 62.0 (59.2-64.8) | 28.2 (25.6-30.8) |
| McDonald, 2012^(28)^ | Cape Town, South Africa | 1998-2006 | HC-II, HPV16/18 (Linear Array) | 1371 | 8050 | Women attending primary or routine HIV care | Cross sectional | 17-65 | 34 (26–38) | NR | NR | 52.4 (49.8-55.1) | 9.3 (7.8-10.9) |
| Mungo, 2021^(29)^ | Kisumu, Kenya | 2019-2020 | Smartphone based cervicography | 92 | - | WLHIV undergoing ccx screening in HIV care and HR-HPV+ | Cross sectional | 25-49 | 37 ±7 | 100% (98% VS) | 449 (±281) | 100% | 15.2% |
| Ndizeye, 2019^(30)^ | Bujumbura, Burundi | 2017 | VIA, Cytology ASCUS+, LSIL+, HSIL+, OncoE6, HPV (PCR); HPV -> OncoE6 or VIA | 679 | - | Women attending routine HIV care | Cross sectional | 25-65 | 44 (37, 52) | NR | NR | 38.2 (34.5-42.0) | 2.8 (1.7-4.3) |
| Paboriboune, 2022^(31)^ | Lao People's Democratic Republic | 2014-2015 | careHPV, Cytology ASCUS+ | 631 | - | Women attending HIV reference centers | Cross sectional | 25-65 | 36 (31,42) | 93% | 374 (240–504) | 35.2% | 7.4% |
| Petry, 1999^(32)^ | Hannover, Germany | 1990-1998 | Cytology HSIL+ | 136 | - | Women attending routine HIV care | Prospective | 19-61 | 33 | NR | NR |  |  |
| Pimple, 2022^(33)^ | Mumbai, India | 2010-2015 | HC-II, Cytology ASCUS+, LSIL=, HSIL+ | 291 | - | WLHIV referred to tertiary cancer care | Retrospective cohort | 30-49 | 36 | 74.2% | NR | 34.4% | 8.6% |
| Raposo, 2011^(34)^ | Rio de Janeiro, Brasil | 1996-2007 | Cytology (ASCUS+), HC-II | 222 | - | Women enrolled in prospective open cohort at FIOCRUZ | Cross sectional | 16-60 | 32 (26-38) | 50.0% | 348 (202-546) | 53.2 (46.4-59.9) | 9.9 (6.3-14.6) |
| Sahasrabuddhe, 2012^(35)^ | Pune, India | 2006-2007 | VIA, Cytology ASCUS+, LSIL+, HSIL+ | 287 | - | WLHIV attending public-sector ART centre | Cross sectional | - | 30 (27, 34) | 26.0% | 343 (241, 497) | * | 11.2 (7.5-15.7) |
| Strickler, 2020^(36)c^ | USA -3 cities (WIHS) | 2013-2015 | Cobas HPV test, Cytology ASCUS+/LSIL+, p16/Ki-67 | 820 | - | Women enrolled in prospective open cohort (WIHS, 37%) and WLHIV referred to colposcopy (63%) | Prospective | - | 46 (±11) | 95.0% | 592 (367-846) | 29%  (screening gp)  42%  (colposcopy gp)  36% (overall) | CIN3+: 5.9% (screening gp)  9.4% (colposcopy)  8.2% (overall) |
| Wang, 2021 | Maryland, USA | NR | HC-II, Cytology ASCUS+ | 71 | - | Women attending routine HIV care | Cross sectional | ≥30 years | 50 (40-56) | 94% | 89% ≥200 cells/µl | 39.7% | 6.3% |
| Womack, 2000^(37)^ | Harare, Zimbabwe | 1999 | HC-II | 249 | 217 | Women attending primary care | Cross sectional | 25-55 | 59% aged 25-34 yrs | NR | NR | 64.3 (58.0-70.2) | 17.3 (12.8-22.5) |
| Zhang, 2014^(38)^ | Yunnan, China | 2009 | HC-II | 83 | - | WLHIV undergoing ccx screening integrated in HIV care | Cross sectional | 18+ | 34 [19-68] | 57.9% | 441 | 48.2 (37.1-59.4) | 8.4 (3.5-16.6) |
| Zhang, 2020^(39)^ | Yunnan, China | 2011-2013 | HC-II | 545 | - | WLHIV attending ART services or identified through VCT centres | Cross sectional | 18+ | 36 (±9) | 77.6% | 499 (±234) | 39.6% | 7.3% |

WLHIV=women living with HIV; NR=not reported; LBC=liquid based cytology; ASCUS+=; LSIL+=; HSIL+=; VIA=; VILI=; DC=digital cervicography; HC-II=Hybrid Capture II; HC-I=Hybrid Capture I; WIHS= Women’s Interagency HIV Study, VS=virally suppressed; ccx=cervical cancer; VCT=voluntary counselling and testing; *taken from separate paper in same cohort

^a^All enrolled women previously tested positive for HR-HPV, this study included in the screening-triage analyses only

^b^The study recruited approximately half of the enrolled women from primary care facilities and community outreach and half of the women from colposcopy clinics referred for cytological abnormalities

^c^ The study recruited one-third of women from routine clinics and two-thirds of women from colposcopy clinics referred for cytological abnormalities (follow-up of an abnormal Pap test, repeat colposcopy for persistent SIL or CIN, or for follow-up of treatment

# Methodological quality of studies evaluating diagnostic accuracy of VIA for CIN2+/CIN3+

The first QUADAS item evaluated if tests were conducted on a representative sample of WLHIV, i.e. women recruited from HIV clinics or attending cervical cancer screening, the majority of whom were taking ART, of whom the majority were HIV virally suppressed with median CD4+ greater than 350 cells/µl (controlling HIV disease). Of 14 studies, 6 scored ‘yes’^(7, 8, 17, 18, 33)^, 3 scored ‘no’ and 5 were ‘unclear’ (Supplementary Figure 3). Studies scoring ‘no’ enrolled women among whom a smaller proportion or none were on ART, or ART users with low CD4+ count^(22, 24, 35)^. Of the 5 studies scoring ‘unclear’, two^(3, 30)^ did not provide any data on HIV related factors and four^(14-16, 24)^ excluded women with evidence of STI, cervicitis or inflammation (which may impact VIA reading; exclusion of these women may be les representative of the wider population of WLHIV). An appropriate reference standard (***Item 3***) was used in all studies (colposcopy with directed biopsies as minimum), although there were differences in item 5 (partial verification bias avoided) and item 6 (differential verification avoided). For the former, 7 of 14 studies scored ‘yes’ (i.e. proportion of women with histology verified CIN2+ was >70%), while 6 scored ‘yes for the latter. Ten of the 14 studies had implemented second independent reading of histopathology. For item 8 (index test well described), 12 of 14 studies described operator (nurse or clinician) and training of VIA operator. In 9 studies, VIA and colposcopy exams were blinded to the other, and two studies reported frequent QC and supervision of VI operators (this was unclear for 10 studies). In 11 studies, there was an acceptable delay between the VIA and colposcopy/biopsy exam (maximum 12 weeks), one study evaluated baseline VIA for cumulative CIN2+ 36 months later^(22)^ and was unclear for 3 studies^(3, 7, 33)^. Uninterpretable results were presented (if occurred) by all 14 studies (item 13).

In 14 studies, the histo-pathologist was blind to the VIA result (item 11), and the VIA operator was blind to the histology diagnosis (item 10). Participant flowcharts and/or participant withdrawal were given in 12 studies (item 14).

Stratified analyses were conducted according to methodological quality of studies using three categories: 1) by the proportion of women with cervical biopsy and histology verification of disease; 2) frequency of training and supervision of VIA operators and 3) participant selection and proportion of women with good HIV control.

Restricting the analysis to five studies^(7, 8, 18, 22, 24)^, for which the proportion of women with biopsy and histological verification was >95%, the pooled sensitivity for CIN2+ was 56.0% (95%CI: 45.4-66.1; *I^2^*=65%) and 65.0% (95%CI: 52.9-75.4; *I^2^=42%*) for CIN3+. In four studies for which the proportion of women with biopsy and histological verification was 50-95%^(5, 18, 40, 41)^, the pooled sensitivity for CIN2+ was 65.1% (95%CI: 52.1-76.1, *I^2^*=59%) and 70.4 (95%CI; 61.4-78.0; *I^2^=1%)* for CIN3+. In five studies for which the proportion of women with biopsy and histological verification was less than 50%^(30, 33, 42-44)^, the pooled sensitivity was 83.9% (78.6-88.2; *I^2^*=6%). The CIN2+ prevalence in these studies was 8.0% (95%CI: 3.4-12.6). In all studies where biopsy rate was 100%, a single training event was conducted at study initiation and further supervision or QA/QC was not documented. Three separate studies^(16, 17, 30)^ documented frequent training, supervision and experience of VIA operators, and although sensitivity for CIN2+ was high (83.8%, 95%CI: 75.9-89.5; *I^2^*=6.3), the biopsy rate in these studies was low; 8%^(30)^, 13%^(17)^ and 23%^(16)^.

We restricted analysis on the association of HIV-related factors and diagnostic accuracy of VIA to those studies in which the biopsy rate was 100%. In two studies^(8, 45)^ that provided diagnostic accuracy estimates according to CD4+ count, the sensitivity of VIA was higher in women with lower CD4+ count (≤350 cells/µl=59.2%, 95%CI: 44.1-72.7 vs. >350 cells/µl=48.4%, 95%CI: 33.9-63.2; Relative Sensitivity of VIA in women ≤350 vs. >350 cells/µl =1.22, 95%CI: 0.96-1.57). There was corresponding lower specificity in women with CD4+ count ≤350 cells/µl vs. >350 cells/µl (60.0%, 95%CI: 43.8-74.3 vs. 71.1%, 95%CI: 56.4-82.3; Relative Specificity=0.84, 95%CI: 0.74-0.96**)**. The CIN2+ prevalence was higher among WLHIV with CD4+ ≤350 cells/µl compared to women with CD4+ >350 cells/µl (29.3% [95%CI: 24.8-33.8] vs. 19.2% [95%CI: 16.1-22.3]) as was the proportion of VIA screen positive women (45.7% [95%CI: 41.0-50.5] vs. 30.6% [95%CI: 27.0-34.2]).

In the same two studies^(8, 45)^, the sensitivity of VIA was higher in women recently initiating ART (<2 years duration ART: 58.8%, 95%CI: 42.6-73.3) compared to women on prolonged duration ART (≥2 years: 46.7%, 95%CI: 30.6-63.6; Relative Sensitivity=1.27, 95%CI: 0.95-1.68**)** corresponding with lower CD4+ count in these women (median 394 and 478 cells/µl, respectively in one study^(45)^). There was also lower specificity in short compared to prolonged duration ART users (67.8% [95%CI: 56.9-77.1] vs. 77.5% [95%CI: 68.6-84.5]; Relative Specificity=0.88, 95%CI: 0.80-97). Pooled sensitivity among ART-naïve women was also higher compared to that in prolonged ART users (55.4%, 95%CI: 38.6-71.1), although not significantly (Relative Sensitivity=1.18, 95%CI: 0.88-1.59). The specificity of VIA was lower in ART-naïve women (Relative specificity=0.86, 95%CI: 0.79-0.94). The CIN2+ prevalence was higher among short-duration ART users compared to prolonged ART duration users or ART-naïve women (23.2%, 14.3% and 18.5%, respectively).

**Supplementary Figure 1. Methodological quality summary for studies evaluating visual inspection: review authors' judgements about each methodological quality item for each included study**

# Methodological quality of studies evaluating diagnostic accuracy of cervical cytology for CIN2+/CIN3+

The first QUADAS item evaluated if tests were conducted on a representative sample of WLHIV, i.e. women recruited from HIV clinics or attending cervical cancer screening, the majority of whom were taking ART, of whom the majority were HIV virally suppressed with median CD4+ greater than 350 cells/µl (controlling HIV disease). Of 23 studies, 9 scored ‘yes’, 12 scored ‘no’ and 2 was ‘unclear’ (Supplementary Figure 4). Of those scoring ‘no’, 9 studies enrolled women in the pre- or early combination ART era^(4, 6, 9, 19, 24, 25, 32, 34, 35, 46)^; in three studies, the study population was enriched for CIN2+^(12, 20, 47)^.

An appropriate reference standard was used in 18 studies (colposcopy with directed biopsies as minimum; partial verification avoided); in 5 studies screen positive women were referred to colposcopy only^(5, 13, 31, 33, 47)^ . There were also differences in proportion of women who underwent biopsy with histological verification : greater than 70% of all enrolled women had at least one biopsy in seven studies (biopsy rate was 100% in 9 seven studies^(4, 6, 8, 9, 12, 13, 20, 24, 25)^, 95% in one study^(45)^ and 74% in one study^(14)^), less than 70% in 7 studies (biopsy rate was 57%^(5)^, 56%^(45)^, 43%^(34)^, 39%^(33)^, 28%^(23)^, 18-32%^(19)^, 19%^(35)^, 16%^(31)^, 13%^(17)^ and 8%^(30)^) and unclear for 2 studies^(32, 47)^.

For item 8 (index test well described), 10 studies^(6, 8, 9, 14, 23-25, 35, 45)^ provided adequate information on conduct of cytology including personnel involved and location of testing but only 8 documented QA/QC, either through second independent reading or enrolment in EQA programmes^(4, 6, 9, 14, 18, 24, 25)^. In 12 studies, there was an acceptable delay between the cytology and colposcopy/biopsy exam (maximum 12 weeks; *item 4*). Uninterpretable results were documented in 16 studies (*item 13*). In 15 studies, cytology and colposcopy/histology endpoint were conducted without knowledge of the other, it was unclear for 7 studies, and in one study colposcopists and pathologists had knowledge of the cytology result^(34)^.

Sixteen studies reported follow-up of participants who entered the study, including the withdrawals (*item 14*); in ten of the studies, the proportion of women who attended all visits and included in final analysis was greater than 80% of those enrolled and in one study, there was a significant proportion lost to follow-up (50%^(34)^).

The main sources of bias in studies evaluating **cervical cytology** was also linked to endpoint ascertainment and participant selection. Restricting the analysis to 6 studies^(4, 6, 8, 24, 25, 45)^ where the proportion of women with biopsy and histological verification was 100%, the sensitivity of HSIL+ for CIN2+ was 51.4% (95%CI: 33.9-68.6; *I^2^=66.0%; data not shown*). Sensitivity of HSIL+ for CIN2+ was lower in studies with low biopsy rate; 32.2% (95%CI: 24.6-40.8; *I^2^=13.7%*) in 6 studies with biopsy rate was less than 70% ^(17, 23, 30, 34, 35, 45)^. Cytology HSIL+ had the highest combined sensitivity and specificity in studies where majority of women had histological verification of disease, were controlling HIV and in settings with External Quality Assessment programmes in place. In three studies with biopsy rate between 74% and 100%^(8, 14, 45)^ and that included mostly women controlling HIV (high proportion of women taking ART with high median CD4+ count), the sensitivity of HSIL+ for CIN2+ was 74.5% (95%CI: 69.4-79.0, *I^2^=15.8%*) and specificity was 89.0% (95%CI: 75.8-95.5, *I^2^=92.2%; data not shown*). The proportion of screen-positive women was 27.9% (95%CI: 19.8-35.9) and pooled PPV was 66.8% (95%CI: 48.3-85.3). Two of these studies^(14, 45)^ conducted in the National Health Laboratory Service, the reference laboratory for cervical screening in South Africa also reported enrolment in regular External Quality Assessment programmes for cytology. While no difference in sensitivity was observed in studies that reported QA/QC of cytology compared to those that did not, or where it was unclear, non-reporting of QA/QC in study methodologies cannot be ruled out.

In five studies^(8, 14, 35, 45)^, sensitivity was higher in women with CD4+ ≤350 cells/µl compared to >350 cells/µl (Relative Sensitivity= 1.16, 95%CI: 1.01-1.34) with no difference in specificity (Relative Specificity=0.96, 95%CI: 0.93-1.00).

**Supplementary Figure 2. Methodological quality summary for studies evaluating cervical cytology: review authors' judgements about each methodological quality item for each included study**

# Methodological quality of studies evaluating diagnostic accuracy of HPV-DNA tests for CIN2+/CIN3+

For Item 1 (representative sample), fourteen (50%) studies enrolled women considered to be controlling HIV^(5, 7, 8, 11, 14, 17, 18, 23, 27, 31, 33, 36, 38, 39)^; 5 studies did not provide any clinical or HIV-related information to assess^(3, 13, 26, 28, 30)^. Of the nine studies scoring ‘no’, six enrolled women in the pre- or early ART era^(9, 10, 19, 22, 34, 37)^, when women were less likely to be controlling HIV infection; three studies enrolled equal numbers of women from screening clinics and colposcopy clinics (women referred for cervical abnormalities) and the study population was enriched for CIN2+^(20, 21, 47)^. An appropriate reference standard (***Item 3***) was used in all studies (colposcopy with directed biopsies as minimum), although there were differences in item 5 (partial verification bias avoided) and item 6 (differential verification avoided).

For item 5, in nineteen (68%) studies, colposcopy was conducted among all women enrolled^(7-10, 17-22, 26, 28, 30, 31, 33, 34, 37-39)^. In nine studies, women who were screen positive were referred to colposcopy. In four of these studies, the proportion of screen positives was >80% (in two studies, women were referred if ASCUS+ or VIA+, and 25% of all women with negative Pap smears and negative VIA were randomly referred to colposcopy resulting in 94% of women undergoing colposcopy^(14, 27)^; in one study, women who screened positive for careHPV, VIA or cytology were referred resulting in 80% of women undergoing colposcopy^(3)^ and in one study women with ASC-US+ or HPV test positive, as well as a random 21% with a normal Pap and negative HPV test were referred to colposcopy^(36)^). For the remaining five studies^(5, 11, 13, 23, 47)^, the proportion of women referred to colposcopy was less than 60% (women screening positive with any of cytology, VIA or HPV-DNA. It is unclear if women who were HPV negative are considered to be low risk of disease and colposcopy-histological verification would not be considered necessary.

For item 6 (differential verification avoided), eight studies scored ‘yes’ (i.e. proportion of women with histology verified CIN2+ was >70%)^(7-9, 14, 18, 20-22, 27)^. Four studies scored ‘no’; the proportion of women undergoing biopsy with histology verification was less than 70% (8%^(30)^, 10%^(11)^ 13%^(17)^, 16%^(31)^, 18%^(19)^, 22%^(13)^, 26%^(38)^, 28%^(23)^, 31%^(36)^, 39%^(33)^ and 43%^(34)^). The proportion of women undergoing biopsy and histology-verification was not reported for nine studies^(3, 5, 10, 22, 26, 28, 37, 39, 47)^ and classified as ‘unclear’.

Twelve (43%) of the 28 studies had implemented second independent reading of histopathology^(3, 10, 11, 14, 17, 18, 21, 22, 26-28, 36)^. For item 8 (index test well described), all but nine studies^(5, 10, 13, 30, 33, 34, 37, 38, 47)^ provided information on HPV test conduct and location of testing. All studies using clinician collected cervical specimen for HPV testing. One study^(27)^ evaluated GeneXpert using clinician collected cervical specimens stored in Digene storage medium. In 11 studies, there was an acceptable delay between the HPV test and colposcopy/biopsy exam (maximum 12 weeks; *item 4*), one study evaluated baseline HC-II for cumulative CIN2+ 36 months later^(22)^ and was unclear for 5 studies^(3, 7, 21, 28, 38)^. In all studies, HPV test and colposcopy/histology endpoint were conducted without knowledge of the other.

In 19 of the studies, the proportion of women who attended all visits and included in final analysis (*item 14*) was greater than 80% of those enrolled and in three studies, there was a significant proportion lost to follow-up (30%^(3)^, 50%^(34)^ and 60% did not attend colposcopy^(10)^).

**Supplementary Figure 3. Methodological quality summary for studies evaluating HPV DNA tests: review authors' judgements about each methodological quality item for each included study**

The main source of bias in studies evaluating **HPV-DNA tests** was linked to participant selection; HPV DNA tests had lower specificity among women with lower CD4+ count or recent ART initiation (and correspondingly lower CD4+ count) linked to higher HPV prevalence among these women. Stratified analyses were conducted according to methodological quality of studies using two categories: 1) by the proportion of women with cervical biopsy and histology verification of disease and 2) participant selection and proportion of women with HIV control (using ART status and CD4+ count). Additional sub-analyses were conducted by age, HIV status and user applied modifications to HPV-DNA tests to evaluate their role on test specificity. In seven studies^(7-9, 14, 21, 27, 45)^ for which the proportion of women with biopsy and histological verification was greater than 70%, the pooled sensitivity was 89.7% (95%CI: 86.7-92.0; *I^2^*=8.8%) and pooled specificity was 52.9% (95%CI: 49.3-56.5; *I^2^*=42.2%) for CIN2+ (*data not shown*). The corresponding estimates in six studies for which the proportion of women with biopsy and histological verification was less than 70% (56%^(45)^, 18-32%^(19)^, 23%^(16)^, 19%^(35)^, 13%^(17)^ and 8%^(30)^) were 94.3% (95%CI: 90.2-96.7; *I^2^*=3.8%) and 60.4% (95%CI: 51.7-68.5; *I^2^*=84.3%), respectively. In four studies,^(18, 41, 48)^ the sensitivity of HPV-DNA was lower in WLHIV with high CD4+ count (CD4+ >350 vs. ≤350 cells/µl Relative Sensitivity= 0.92, 95%CI: 0.87-0.98) but with higher specificity (61.1% vs. 42.0%; Relative Specificity=1.45, 95%CI: 1.32-1.61; Figure 5 and Supplementary Figure 6). Similar findings were observed when using 500 cells/µl as threshold (CD4+ <500 vs. ≤500 cells/µl: Relative Sensitivity=0.92, 95%CI: 0.82-1.03; RSpec=1.29, 95%CI: 1.17-1.43). In three studies^(8, 45)^, the sensitivity of HPV-DNA tests was non-significantly higher in women recently initiating ART compared to women on prolonged duration ART (<2 years vs. ≥2 years: 90.9%, 95%CI: 82.9-95.3 vs. 83.9%, 95%CI: 73.6-90.7; Relative Sensitivity=1.07, 95%CI: 0.96-1.19) and specificity was 30% lower (46.4%, 95%CI: 39.0-54.0 vs. 65.4%, 95%CI: 58.6-71.7; Relative Specificity=0.71, 95%CI: 0.61-0.81; **Figure 5 and Supplementary Table 5**), corresponding with higher HR-HPV prevalence associated with lower CD4+ count in women recently initiating ART. Pooled sensitivity among ART-naïve women was similar to that among women on prolonged ART (Relative Sensitivity=1.05, 95%CI: 0.92-1.18), but as for recent ART initiators, specificity was lower in ART-naïve women compared to prolonged ART users (**Relative Specificity=0.74, 95%CI: 0.65-0.84**), possibly linked to higher HIV viral load compared to prolonged ART users, although CD4+ count was higher than short-duration ART users. One study among 1238 WLHIV in two countries (Burkina Faso and South Africa) provided diagnostic accuracy estimates for HC-II according to HIV-1 viral suppression among ART users^(45)^. Among 384 WLHIV taking ART in Burkina Faso, 87% of whom had suppressed HIV-1 VL (<1000 copies/ml), there was no difference in sensitivity of HC-II in women who had HIV-1 viral suppression or not (HIV-1 VL <1000 vs. ≥1000 copies/ml: 95.5%, 95%CI: 77.2-99.9 vs. 100.0, 95%CI: 39.8-100.0), but specificity was higher in women with suppressed HIV-1 VL (66.7%, 95%CI: 61.1-71.9 vs. 58.7%, 95%CI: 43.2-73.0). Findings were similar in 368 WLHIV taking ART in South Africa, 81% of whom were virally suppressed (sensitivity :HIV-1 VL <1000 vs. ≥1000 copies/ml: 90.5%, 95%CI: 80.4-96.4 vs. 93.3, 95%CI: 68.1-99.8; specificity: 52.1%, 95%CI: 45.5-58.7 vs. 33.9%, 95%CI: 21.8-47.8).

# Supplementary Table 2. Meta-analysis of diagnostic accuracy of VIA-DC and VILI for CIN2+ and CIN3+ among WLHIV

|  | **N populations** | **N WLHIV** | **CIN2+ prevalence,**  **% (95%CI)** | **Test positive,**  **% (95%CI)** | **Sensitivity**  **(%, 95%CI)** | ***I^2^*** | **Specificity**  **(%, 95%CI)** | ***I^2^*** |
| --- | --- | --- | --- | --- | --- | --- | --- | --- |
| **CIN2+** |  |  |  |  |  |  |  |  |
| VIA – Digital cervicography^(4, 7, 14)^ | 3 | 1695 | 21.0 (15.0-27.1) | 37.8 (22.1-53.5) | 73.0 (59.9-83.0) | *70.9%* | 73.0 (55.2-85.6) | *95.4%* |
| VILI ^(15, 17, 18)^ | 4 | 2586 | 11.1 (4.9-17.3) | 26.7 (15.8-37.6) | 74.2 (58.5-85.5) | *77.4%* | 79.3 (70.2-86.2) | *94.4%* |
| Combination of VIA and VILI*^(17, 18)^ | 3 | 2257 | 11.0 (3.3-18.7) | 24.4 (4.7-44.1) | 69.1 (57.2-78.9) | *59.3%* | 84.3 (61.0-94.9) | *98.3%* |
| **CIN3+** |  |  |  |  |  |  |  |  |
| VIA – Digital cervicography^(4, 7, 14)^ | 3 | 1695 | 9.1 (7.7-10.4) | 38.1 (22.1-54.1) | 77.3 (68.5-84.2) | *9.5%* | 68.4 (48.0-83.6) | *96.7%* |
| VILI ^(17, 18)^ | 3 | 2256 | 4.8 (1.8-7.8) | 25.4 (12.2-38.7) | 80.4 (61.8-91.2) | *59.5%* | 78.3 (65.5-87.2) | *96.8%* |
| Combination of VIA and VILI*^(18)^ | 2 | 1129 | 3.9 (2.8-5.0) | 31.6 (28.9-34.3) | 69.7 (57.6-79.5) | *-* | 69.4 (66.6-72.1) | *-* |
|  |  |  |  |  |  |  |  |  |

VIA-DC=visual inspection, acetic acid with digital cervicography; VILI-visual inspection with lugol’s iodine; VIA or VILI positive=screen positive if abnormal on either VIA or VILI

# Supplementary Figure 4. Meta-analysis of performance of HPV tests for CIN2+ (top panel) and CIN3+ (bottom panel) among WLHIV by restricting targeted genotype approach and threshold in 4 studies

*low threshold= manufacturer defined threshold; Three studies representing four populations provided diagnostic accuracy data according to different test positivity thresholds; two for HC-II (from 1 relative light unit [RLU] to 20RLU; RLU corresponding with higher HPV viral load)^(37, 45)^ and one study for GeneXpert (modification of PCR cycle thresholds; a low cycle threshold corresponds with a high HPV viral load)^(21)^. For Kelly, 2021 (SA and BF)^(18)^, HC-II targets 13 HR types (HPV16, 18, 31, 33, 35, 39, 45, 51, 52, 56, 58, 59, 68); 8HR positivity was defined as being positive for both HC-II and any of the 8HR types (HPV16, 18, 31, 33, 35, 45, 52, or 58) using INNO-LiPA genotyping assay, low threshold is based on threshold of 1 relative light unit [RLU] and high threshold corresponds with 20RLU. For Kuhn, 2020^(21)^, GeneXpert 5-channel targets 14 HR types (HPV16, 18, 31, 33, 35, 39, 45, 51, 52, 56, 58, 59, 66, 68) and 3-channel targets 8HR (HPV16, 18, 31, 33, 35, 45, 52, or 58); low threshold corresponds with a high cycle threshold value (equivalent to a low viral load; many cycles needed to become positive) and a high threshold corresponds with a low cycle threshold value (equivalent to a high viral load; few cycles needed to become positive). For Chung, 2013^(8)^, GP5+/6+ was used to define 14 HR positive (HPV16, 18, 31, 33, 35, 39, 45, 51, 52, 56, 58, 59, 66, 68) and 8HR positive (HPV16, 18, 31, 33, 35, 45, 52, or 58), there was no variation in threshold for test positivity.

Three studies provided evaluated different test positivity thresholds; two for HC-II (from 1 relative light unit [RLU] to 20RLU; RLU corresponding with higher HPV viral load)^(37)^, one study for GeneXpert (modification of PCR cycle thresholds; a low cycle threshold corresponds with a high HPV viral load)^(21)^. Using the standard 14-HR (low threshold) approach, the pooled test positivity in the 3 studies was 54.0% (95%CI: 45.2-62.7) compared with 45.4% (95%CI: 34.9-55.9) test positivity using the 8HR (low threshold) approach and 41.3% (95%CI: 38.5-44.1) using 8-HR (high threshold) approach.

Specificity increased with the high compared to low threshold (Relative Specificity=1.32, 95%CI: 1.22-1.43) but at the expense of reduced sensitivity (Relative Sensitivity=0.90, 95%CI: 0.83-0.97). Combining a restricted 8-HR approach with a high threshold increased the specificity further in three studies^(18, 21)^ (76.6%, 95%CI: 71.9-80.7; Relative Specificity vs. 13-HR approach [low threshold]=1.36, 95%CI: 1.26-1.47) but with loss in sensitivity for CIN2+ (79.7%; Relative Sensitivity=0.89, 95%CI: 0.82-0.97) and CIN3+ (83.6%, 95%CI: 70.2-91.7; Relative Sensitivity=0.89, 95%CI: 0.80-1.00).

# Supplementary Figure 5. Meta-analysis of diagnostic accuracy of triage of HR-HPV positive women for CIN2+ (A) and CIN3+ (B) among WLHIV in 6 studies

(A)

(B)

# Supplementary Table 3. Diagnostic accuracy of screening tests (HPV-DNA, VIA and cytology) by age group in three studies

|  | **N populations** | **N women** | **CIN2+ prevalence,**  **% (95%CI)** | **Test positive,**  **% (95%CI)** | **Sensitivity**  **(%, 95%CI)** | ***I^2^*** | **Relative Sensitivity (95%CI)** | **Specificity**  **(%, 95%CI)** | ***I^2^*** | **Relative Specificity (95%CI)** |
| --- | --- | --- | --- | --- | --- | --- | --- | --- | --- | --- |
| **HPV-DNA** |  |  |  |  |  |  |  |  |  |  |
| 20-24 years | 1 | 11 | 9.1 (0.2-41.3) | 63.6 (30.8-89.1) | 100.0 (2.5-100.0) | *-* |  | 40.0 (12.2-73.8) | *-* |  |
| 25-29 years | 3 | 282 | 17.3 (-1.0-36.2) | 58.9 (47.7-70.2) | 79.6 (63.5-89.7) | *3.3%* | 1.00 | 44.4 (36.2-52.8) | *32.0%* | 1.00 |
| 30-34 years | 3 | 399 | 15.6 (0.4-30.7) | 54.6 (44.6-64.7) | 85.4 (72.6-92.8) | *18.0%* | 1.09 (0.90-1.31) | 50.6 (43.0-58.1) | *3.4%* | 1.14 (0.95-1.36) |
| 35-39 years | 3 | 391 | 16.9 (8.4-25.4) | 49.8 (38.9-60.6) | 92.8 (83.3-97.1) | *16.3%* | 1.13 (0.96-1.34) | 59.0 (51.4-66.3) | *37.9%* | 1.34 (1.12-1.60) |
| 40-44 years | 3 | 319 | 17.1 (5.3-28.9) | 47.1 (34.0-60.1) | 89.3 (77.7-95.2) | *3.1%* | 1.08 (0.91-1.29) | 61.8 (53.8-69.3) | *57.1%* | 1.38 (1.15-1.66) |
| 45-50 years | 3 | 175 | 16.9 (1.3-32.5) | 44.5 (37.2-51.9) | 86.9 (69.4-95.1) | *0.0%* | 1.08 (0.87-1.34) | 63.0 (53.2-71.8) | *0.0%* | 1.42 (1.17-1.73) |
| 51 years+ | 1 | 15 | 33.3 (11.8-61.6) | 40.0 (16.3-67.7) | 60.0 (14.7-94.7) | *-* | 0.71 (0.31-1.61) | 70.0 (34.8-93.3) | *-* | 1.53 (0.95-2.44) |
|  | **N populations** | **N women** | **CIN2+ prevalence,**  **% (95%CI)** | **Test positive,**  **% (95%CI)** | **Sensitivity**  **(%, 95%CI)** | ***I^2^*** | **Relative Sensitivity (95%CI)** | **Specificity**  **(%, 95%CI)** | ***I^2^*** | **Relative Specificity (95%CI)** |
| **VIA** |  |  |  |  |  |  |  |  |  |  |
| 20-24 years | 1 | 11 | 9.1 (0.2-41.3) | 27.3 (6.0-61.0) | 0.0 (0.0-97.5) | *-* | - | 70.0 (34.8-93.3) | *-* | 1.05 (0.72-1.53) |
| 25-29 years | 3 | 283 | 17.3 (-1.0-36.2) | 34.7 (21.9-47.5) | 59.4 (39.5-76.7) | *18.3%* | 1.00 | 70.1 (59.6-78.8) | *25.4%* | 1.00 |
| 30-34 years | 3 | 404 | 15.6 (0.4-30.7) | 31.5 (18.6-44.5) | 53.6 (35.1-71.1) | *26.5%* | 0.90 (0.64-1.27) | 73.1 (63.7-80.8) | *53.5%* | 1.04 (0.93-1.16) |
| 35-39 years | 3 | 389 | 16.9 (8.4-25.4) | 28.6 (16.6-40.6) | 56.7 (38.3-73.5) | *57.5%* | 0.93 (0.68-1.29) | 77.6 (68.9-84.4) | *50.2%* | 1.11 (0.99-1.23) |
| 40-44 years | 3 | 321 | 17.1 (5.3-28.9) | 25.4 (15.5-35.2) | 35.6 (19.9-55.2) | *7.5%* | 0.63 (0.40-1.00) | 77.5 (68.4-84.6) | *29.4%* | 1.10 (0.99-1.23) |
| 45-50 years | 3 | 174 | 16.9 (1.3-32.5) | 29.6 (18.6-40.5) | 41.5 (21.6-64.6) | *0.0%* | 0.80 (0.48-1.32) | 74.8 (63.7-83.4) | *0.0%* | 1.05 (0.92-1.19) |
| 51 years+ | 1 | 13 | 30.8 (9.1-61.4) | 15.4 (1.9-45.4) | 25.0 (0.6-80.6) | *-* | 0.26 (0.03-2.57) | 88.9 (51.8-99.7) | *-* | 1.29 (1.03-1.60) |
|  | **N populations** | **N women** | **CIN2+ prevalence,**  **% (95%CI)** | **Test positive,**  **% (95%CI)** | **Sensitivity**  **(%, 95%CI)** | ***I^2^*** | **Relative Sensitivity (95%CI)** | **Specificity**  **(%, 95%CI)** | ***I^2^*** | **Relative Specificity (95%CI)** |
| **Cytology ASCUS+** |  |  |  |  |  |  |  |  |  |  |
| 20-24 years* | 1 | 11 |  |  |  |  |  |  |  |  |
| 25-29 years | 3 | 276 | 16.9 (-1.7-35.5) | 58.5 (8.1-100.0) | 91.2 (39.7-99.4) | *3.0%* | 1.00 | 39.4 (10.4-78.4) | *89.6%* | 1.00 |
| 30-34 years | 3 | 388 | 16.0 (0.4-31.7) | 62.3 (19.78-100) | 95.3 (55.8-99.7) | *15.3%* | 1.04 (0.89-1.22) | 35.3 (9.0-75.2) | *92.4%* | 0.89 (0.67-1.19) |
| 35-39 years | 3 | 381 | 16.8 (8.0-25.6) | 58.9 (11.6-100) | 97.0 (66.7-99.8) | *0.0%* | 1.03 (0.88-1.22) | 42.3 (11.7-80.3) | *93.0%* | 1.07 (0.81-1.42) |
| 40-44 years | 3 | 310 | 16.7 (4.8-28.6) | 58.9 (23.4-94.4) | 98.3 (75.9-99.9) | *9.5%* | 1.07 (0.87-1.30) | 44.8 (12.7-81.9) | *90.5%* | 1.13 (0.86-1.47) |
| 45-50 years | 3 | 169 | 17.3 (1.6-33.1) | 60.6 (18.9-100) | 95.1 (54.1-99.7) | *15.5%* | 1.04 (0.89-1.22) | 40.3 (10.6-79.3) | *84.7%* | 1.01 (0.73-1.40) |
| 51 years+* | 1 | 13 |  |  |  |  |  |  |  |  |
|  | **N populations** | **N women** | **CIN2+ prevalence,**  **% (95%CI)** | **Test positive,**  **% (95%CI)** | **Sensitivity**  **(%, 95%CI)** | ***I^2^*** | **Relative Sensitivity (95%CI)** | **Specificity**  **(%, 95%CI)** | ***I^2^*** | **Relative Specificity (95%CI)** |
| **Cytology HSIL+**** |  |  |  |  |  |  |  |  |  |  |
| 20-24 years* | 1 | 11 |  |  |  |  |  |  |  |  |
| 25-29 years | 2 | 182 | 25.6 (19.3-32.0) | 21.5 (15.7-27.3) | 57.3 (42.6-70.9) | *-* | 1.00 | 91.5 (73.3-97.7) | *-* | 1.00 |
| 30-34 years | 2 | 261 | 23.3 (18.2-28.4) | 21.5 (15.7-27.3) | 68.8 (55.9-79.3) | *-* | 1.21 (0.89-1.65) | 90.3 (71.0-97.3) | *-* | 0.99 (0.94-1.04) |
| 35-39 years | 2 | 250 | 21.1 (16.0-26.1) | 26.4 (21.0-31.8) | 84.9 (72.6-92.3) | *-* | 1.45 (1.10-1.92) | 90.9 (72.4-97.5) | *-* | 0.99 (0.94-1.04) |
| 40-44 years | 2 | 199 | 20.4 (14.9-25.9) | 24.1 (18.1-30.0) | 70.5 (55.4-82.2) | *-* | 1.22 (0.88-1.71) | 92.1 (74.9-97.9) | *-* | 1.01 (0.95-1.07) |
| 45-50 years | 2 | 100 | 24.2 (16.0-32.5) | 20.9 (12.9-28.9) | 69.3 (49.2-84.1) | *-* | 1.28 (0.86-1.90) | 96.7 (83.9-99.4) | *-* | 1.04 (0.95-1.14) |
| 51 years+* | 1 | 13 |  |  |  |  |  |  |  |  |

PPV=positive predictive value; NPV=negative predictive value (1-NPV is the proportion of false negative among test negative); *insufficient data to calculate; ** excluded ^(18)^ Burkina Faso estimates due to poor performance at threshold HSIL+

# Supplementary Table 4. Pooled relative sensitivity and relative specificity of HPV testing screening compared to VIA and cervical cytology for detection of CIN2+, stratified by age

|  | **N studies** | **Relative Sensitivity (95%CI)** | **p-value** | **Relative Specificity (95%CI)** | **p-value** |
| --- | --- | --- | --- | --- | --- |
| HPV vs. VIA |  |  |  |  |  |
| 20-24 years | 1 | * |  | 0.57 (0.24- 1.35) | 0.20 |
| 25-29 years | 3 | 1.31 (1.00-1.73) | 0.05 | 0.62 (0.52-0.74) | <0.001 |
| 30-34 years | 3 | 1.59 (1.22-2.06) | <0.001 | 0.68 (0.59-0.78) | <0.001 |
| 35-39 years | 3 | 1.56 (1.15-2.10) | <0.001 | 0.76 (0.67-0.86) | <0.001 |
| 40-44 years | 3 | 2.14 (1.54-2.98) | <0.001 | 0.80 (071-0.90) | <0.001 |
| 45-50 years | 3 | 1.73 (1.18-2.55) | 0.01 | 0.86 (0.73-1.00) | 0.05 |
| 51 years+ | 1 | 2.40 (0.38-15.14) | 0.35 | 0.79 (0.49-1.26) | 0.32 |
|  |  |  |  |  |  |
| HPV vs. Cytology ASCUS+ |  |  |  |  |  |
| 20-24 years | 1 | 1.00 |  | 0.80 (0.30-2.13) | 0.66 |
| 25-29 years | 3 | 0.82 (0.67-1.00) | 0.05 | 1.02 (0.81-1.27) | 0.88 |
| 30-34 years | 3 | 0.85 (0.68-1.06) | 0.16 | 1.27 (1.05-1.55) | 0.02 |
| 35-39 years | 3 | 0.99 (0.89-1.09) | 0.78 | 1.28 (1.05-1.56) | 0.01 |
| 40-44 years | 3 | 0.93 (0.83-1.03) | 0.16 | 1.28 (1.05-1.57) | 0.02 |
| 45-50 years | 3 | 0.96 (0.80-1.16) | 0.69 | 1.23 (0.98-1.56) | 0.08 |
| 51 years+ | 1 | 0.60 (0.29-1.23) | 0.16 | 1.40 (0.67-2.94) | 0.37 |
|  |  |  |  |  |  |
| HPV vs. Cytology HSIL+ |  |  |  |  |  |
| 20-24 years | 1 | 1.00 |  | 0.40 (0.19-0.85) | 0.02 |
| 25-29 years | 2 | 1.34 (1.01-1.79) | 0.04 | 0.48 (0.34-0.68) | <0.001 |
| 30-34 years | 2 | 1.26 (1.00-1.58) | 0.05 | 0.56 (0.47-0.67) | <0.001 |
| 35-39 years | 2 | 1.07 (0.93-1.23) | 0.36 | 0.62 (0.53-0.71) | <0.001 |
| 40-44 years | 2 | 1.24 (0.99-1.55) | 0.07 | 0.64 (0.51-0.80) | <0.001 |
| 45-50 years | 2 | 1.22 (0.90-1.66) | 0.20 | 0.69 (0.58-0.81) | <0.001 |
| 51 years+ | 1 | 0.75 (0.32-1.74) | 0.50 | 0.78 (0.49-1.23) | 0.28 |

*numbers too small to calculate

# Supplementary Table 5. Diagnostic accuracy of screening tests (HPV-DNA, VIA and cytology) by ART status group in three studies

|  | **N populations** | **N women** | **CIN2+ prevalence,**  **% (95%CI)** | **Test positive,**  **% (95%CI)** | **Sensitivity**  **(%, 95%CI)** | ***I^2^*** | **Relative Sensitivity (95%CI)** | **Specificity**  **(%, 95%CI)** | ***I^2^*** | **Relative Specificity (95%CI)** |
| --- | --- | --- | --- | --- | --- | --- | --- | --- | --- | --- |
| **HPV-DNA** |  |  |  |  |  |  |  |  |  |  |
| ART ≥ 2 years | 3 | 607 | 14.1 (4.6-23.5) | 41.4 (32.7-50.1) | 83.9 (73.6-90.7) | *1.7%* | 0.96 (0.85-1.08) | 65.4 (58.6-71.7) | *44.9%* | 1.35 (1.19-1.53) |
| ART <2 years | 3 | 481 | 21.6 (6.2-36.9) | 61.3 (44.1-78.4) | 90.9 (82.9-95.3) | *44.0%* | 1.04 (0.94-1.15) | 46.4 (39.0-54.0) | *82.8%* | 0.96 (0.82-1.11) |
| ART-naïve | 3 | 458 | 17.0 (1.8-32.3) | 57.9 (53.4-62.4) | 87.6 (77.0-93.7) | *1.5%* | 1.00 | 48.5 (41.0-56.1) | *0.7%* | 1.00 |
|  | **N populations** | **N women** | **CIN2+ prevalence,**  **% (95%CI)** | **Test positive,**  **% (95%CI)** | **Sensitivity**  **(%, 95%CI)** | ***I^2^*** | **Relative Sensitivity (95%CI)** | **Specificity**  **(%, 95%CI)** | ***I^2^*** | **Relative Specificity (95%CI)** |
| **VIA** |  |  |  |  |  |  |  |  |  |  |
| ART ≥ 2 years | 3 | 608 | 14.0 (4.6-23.4) | 25.0 (17.1-32.9) | 40.9 (27.3-56.1) | *49.5%* | 0.81 (0.57-1.14) | 77.9 (70.3-84.1) | *44.8%* | 1.08 (0.99-1.17) |
| ART <2 years | 3 | 484 | 21.5 (6.0-37.0) | 33.9 (18.3-49.6) | 57.7 (43.3-70.9) | *29.1%* | 1.14 (0.87-1.51) | 73.2 (64.4-80.5) | *84.3%* | 1.01 (0.93-1.10) |
| ART-naïve | 3 | 462 | 17.0 (1.7-32.3) | 31.2 (19.9-42.5) | 50.5 (35.7-65.3) | *21.3%* | 1.00 | 72.4 (63.5-79.8) | *58.1%* | 1.00 |
|  | **N populations** | **N women** | **CIN2+ prevalence,**  **% (95%CI)** | **Test positive,**  **% (95%CI)** | **Sensitivity**  **(%, 95%CI)** | ***I^2^*** | **Relative Sensitivity (95%CI)** | **Specificity**  **(%, 95%CI)** | ***I^2^*** | **Relative Specificity (95%CI)** |
| **Cytology ASCUS+** |  |  |  |  |  |  |  |  |  |  |
| ART ≥ 2 years | 3 | 592 | 14.1 (4.4-23.8) | 56.6 (12.9-100.2) | 95.0 (61.4-99.6) | *54.0%* | 1.00 (0.934-1.07) | 45.3 (12.9-82.2) | *97.8%* | 1.13 (0.92-1.40) |
| ART <2 years | 3 | 476 | 21.4 (5.6-37.2) | 65.1 (25.4-105.9) | 97.5 (75.0-99.8) | 10.4% | 1.02 (0.95-1.11) | 34.0 (8.4-74.3) | 96.5% | 0.85 (0.66-1.08) |
| ART-naïve | 3 | 478 | 16.8 (1.2-32.3) | 56.9 (11.2-106.6) | 95.2 (60.6-99.6) | 4.2% | 1.00 | 40.0 (10.6-78.9) | 97.0% | 1.00 |
|  | **N populations** | **N women** | **CIN2+ prevalence,**  **% (95%CI)** | **Test positive,**  **% (95%CI)** | **Sensitivity**  **(%, 95%CI)** | ***I^2^*** | **Relative Sensitivity (95%CI)** | **Specificity**  **(%, 95%CI)** | ***I^2^*** | **Relative Specificity (95%CI)** |
| **Cytology HSIL+**** |  |  |  |  |  |  |  |  |  |  |
| ART ≥ 2 years | 3 | 592 | 14.1 (4.4-23.8) | 15.0 (3.0-27.1) | 61.3 (35.2-82.2) | *48.9%* | 1.51 (1.02-2.23) | 95.6 (87.2-98.5) | *78.9%* | 1.02 (0.99-1.06) |
| ART <2 years | 3 | 476 | 21.4 (5.6-37.2) | 23.2 (1.5-44.9) | 70.8 (45.6-87.6) | *74.5%* | 1.74 (1.13-2.67) | 93.4 (81.8-97.8) | *82.9%* | 1.00 (0.97-1.03) |
| ART-naïve | 3 | 478 | 16.8 (1.2-32.3) | 16.0 (0.4-31.6) | 40.7 (19.0-66.8) | *73.1%* | 1.00 | 93.6 (82.2-97.8) | *82.3%* | 1.00 |

# Supplementary Figure 6. Meta-analysis of diagnostic accuracy of HPV-DNA tests for CIN2+ (panel A and B) and CIN3+ (panel C and D) by CD4+ count in five studies

Relative Sensitivity (RSens) of HPV-DNA for CIN2+ detection in WLHIV with high vs. low CD4+ count (>350 vs. ≤350 cells/µl) = 0.92, 95%CI: 0.87-0.98, p=0.013; Relative Specificity (RSpec)= 1.45, 95%CI: 1.32-1.61, p<0.001

B)

Relative Sensitivity of HPV-DNA for CIN2+ detection in WLHIV with high vs. low CD4+ count= RSens (>500 vs. ≤500 cells/µl) = 0.92, 95%CI: 0.82-1.03, p=0.14; RSpec=1.29, 95%CI: 1.17-1.43, p<0.001

(C)

Relative Sensitivity of HPV-DNA for CIN3+ detection in WLHIV with high vs. low CD4+ count (>350 vs. ≤350 cells/µl) =0.97, 95%CI:0.89-1.06, p=0.54; Rspec=1.47, 95%CI: 1.28-1.68, p<0.001

(D)

Relative Sensitivity of HPV-DNA for CIN3+ detection in WLHIV with high vs. low CD4+ count (>500 vs. ≤500 cells/µl) = 0.85, 95%CI: 0.72-1.01 , p=0.06; RSpec= 1.37, 95%CI: 1.25-1.50, p<0.001

# PRISMA 2020 Checklist

| **Section and Topic** | **Item #** | **Checklist item** | **Location where item is reported** |
| --- | --- | --- | --- |
| **TITLE** | | |  |
| Title | 1 | Identify the report as a systematic review. | Title, page 6: line 32 |
| **ABSTRACT** | | |  |
| Abstract | 2 | See the PRISMA 2020 for Abstracts checklist. |  |
| **INTRODUCTION** | | |  |
| Rationale | 3 | Describe the rationale for the review in the context of existing knowledge. | Page 5: line 17; Page 6: line 28 |
| Objectives | 4 | Provide an explicit statement of the objective(s) or question(s) the review addresses. | Page 6: line 32 |
| **METHODS** | | |  |
| Eligibility criteria | 5 | Specify the inclusion and exclusion criteria for the review and how studies were grouped for the syntheses. | Page 6: line 48 |
| Information sources | 6 | Specify all databases, registers, websites, organisations, reference lists and other sources searched or consulted to identify studies. Specify the date when each source was last searched or consulted. | Page 6: line 42 |
| Search strategy | 7 | Present the full search strategies for all databases, registers and websites, including any filters and limits used. | S1 Appendix |
| Selection process | 8 | Specify the methods used to decide whether a study met the inclusion criteria of the review, including how many reviewers screened each record and each report retrieved, whether they worked independently, and if applicable, details of automation tools used in the process. | Page 6: line 45 |
| Data collection process | 9 | Specify the methods used to collect data from reports, including how many reviewers collected data from each report, whether they worked independently, any processes for obtaining or confirming data from study investigators, and if applicable, details of automation tools used in the process. | Page 7: line 80 |
| Data items | 10a | List and define all outcomes for which data were sought. Specify whether all results that were compatible with each outcome domain in each study were sought (e.g. for all measures, time points, analyses), and if not, the methods used to decide which results to collect. | Page 6: line 48, line 58 |
|  | 10b | List and define all other variables for which data were sought (e.g. participant and intervention characteristics, funding sources). Describe any assumptions made about any missing or unclear information. | Page 8, line 90 |
| Study risk of bias assessment | 11 | Specify the methods used to assess risk of bias in the included studies, including details of the tool(s) used, how many reviewers assessed each study and whether they worked independently, and if applicable, details of automation tools used in the process. | Page 8, line 102 |
| Effect measures | 12 | Specify for each outcome the effect measure(s) (e.g. risk ratio, mean difference) used in the synthesis or presentation of results. | Page 8, line 84 |
| Synthesis methods | 13a | Describe the processes used to decide which studies were eligible for each synthesis (e.g. tabulating the study intervention characteristics and comparing against the planned groups for each synthesis (item #5)). |  |
|  | 13b | Describe any methods required to prepare the data for presentation or synthesis, such as handling of missing summary statistics, or data conversions. | N/A |
|  | 13c | Describe any methods used to tabulate or visually display results of individual studies and syntheses. | Page 8, line 94 |
|  | 13d | Describe any methods used to synthesize results and provide a rationale for the choice(s). If meta-analysis was performed, describe the model(s), method(s) to identify the presence and extent of statistical heterogeneity, and software package(s) used. | Page 8, line 84 |
|  | 13e | Describe any methods used to explore possible causes of heterogeneity among study results (e.g. subgroup analysis, meta-regression). | Page 8, line 104 |
|  | 13f | Describe any sensitivity analyses conducted to assess robustness of the synthesized results. | Page 8, line 104 |
| Reporting bias assessment | 14 | Describe any methods used to assess risk of bias due to missing results in a synthesis (arising from reporting biases). |  |
| Certainty assessment | 15 | Describe any methods used to assess certainty (or confidence) in the body of evidence for an outcome. |  |
| **RESULTS** | | |  |
| Study selection | 16a | Describe the results of the search and selection process, from the number of records identified in the search to the number of studies included in the review, ideally using a flow diagram. | Page 9, line 116; Figure 1 |
|  | 16b | Cite studies that might appear to meet the inclusion criteria, but which were excluded, and explain why they were excluded. | Figure 1 |
| Study characteristics | 17 | Cite each included study and present its characteristics. | S1 Table |
| Risk of bias in studies | 18 | Present assessments of risk of bias for each included study. | S3, S4, S5 Figure |
| Results of individual studies | 19 | For all outcomes, present, for each study: (a) summary statistics for each group (where appropriate) and (b) an effect estimate and its precision (e.g. confidence/credible interval), ideally using structured tables or plots. | Table 1 |
| Results of syntheses | 20a | For each synthesis, briefly summarise the characteristics and risk of bias among contributing studies. | Page 18, line 237 |
|  | 20b | Present results of all statistical syntheses conducted. If meta-analysis was done, present for each the summary estimate and its precision (e.g. confidence/credible interval) and measures of statistical heterogeneity. If comparing groups, describe the direction of the effect. | Table 2, Table 3, Table 4 |
|  | 20c | Present results of all investigations of possible causes of heterogeneity among study results. | Page 18, line 237 |
|  | 20d | Present results of all sensitivity analyses conducted to assess the robustness of the synthesized results. | Page 18, line 237 |
| Reporting biases | 21 | Present assessments of risk of bias due to missing results (arising from reporting biases) for each synthesis assessed. |  |
| Certainty of evidence | 22 | Present assessments of certainty (or confidence) in the body of evidence for each outcome assessed. |  |
| **DISCUSSION** | | |  |
| Discussion | 23a | Provide a general interpretation of the results in the context of other evidence. | Page 19, line 270 |
|  | 23b | Discuss any limitations of the evidence included in the review. | Page 21, line 337 |
|  | 23c | Discuss any limitations of the review processes used. | Page 21, line 337 |
|  | 23d | Discuss implications of the results for practice, policy, and future research. | Page 22, line 353 |
| **OTHER INFORMATION** | | |  |
| Registration and protocol | 24a | Provide registration information for the review, including register name and registration number, or state that the review was not registered. | Page 9, line 109 |
|  | 24b | Indicate where the review protocol can be accessed, or state that a protocol was not prepared. | Page 9, line 109 |
|  | 24c | Describe and explain any amendments to information provided at registration or in the protocol. | n/a |
| Support | 25 | Describe sources of financial or non-financial support for the review, and the role of the funders or sponsors in the review. | Page 23 , first para |
| Competing interests | 26 | Declare any competing interests of review authors. | Page 23, para 2 |
| Availability of data, code and other materials | 27 | Report which of the following are publicly available and where they can be found: template data collection forms; data extracted from included studies; data used for all analyses; analytic code; any other materials used in the review. | All available at Mendeley online repository at  DOI: 10.17632/cn53hzsh5p.2. |

*From:*  Page MJ, McKenzie JE, Bossuyt PM, Boutron I, Hoffmann TC, Mulrow CD, et al. The PRISMA 2020 statement: an updated guideline for reporting systematic reviews. BMJ 2021;372:n71. doi: 10.1136/bmj.n71

For more information, visit: <http://www.prisma-statement.org/>

# References

1. Koliopoulos G, Nyaga VN, Santesso N, Bryant A, Martin-Hirsch PP, Mustafa RA, et al. Cytology versus HPV testing for cervical cancer screening in the general population. Cochrane Database Syst Rev. 2017;8(8):CD008587-CD.

2. Reitsma JB, Rutjes AWS, Whiting P, Vlassov VV, Leeflang MMG, JJ. D. Chapter 9: Assessing methodological quality. . In: Deeks JJ BP, Gatsonis C (editors), editor. Cochrane Handbook for Systematic Reviews of Diagnostic Test Accuracy Version 100 The Cochrane Collaboration, 2009 Available from: <http://srdta.cochrane.org/.2009>.

3. Bansil P, Lim J, Byamugisha J, Kumakech E, Nakisige C, Jeronimo JA. Performance of Cervical Cancer Screening Techniques in HIV-Infected Women in Uganda. Journal of Lower Genital Tract Disease.19(3):215-9.

4. Bateman AC, Parham GP, Sahasrabuddhe VV, Mwanahamuntu MH, Kapambwe S, Katundu K, et al. Clinical performance of digital cervicography and cytology for cervical cancer screening in HIV-infected women in Lusaka, Zambia. Journal of Acquired Immune Deficiency Syndromes: JAIDS.67(2):212-5.

5. Boddu A, Bhatla N, Vashist S, Mathur S, Mahey R, Natarajan J, et al. Cervical Cancer Screening in HIV-Positive Women in India: Why, When and How? J Obstet Gynaecol India. 2021;71(3):304-12.

6. Branca M, Rossi E, Alderisio M, Migliore G, Morosini PL, Vecchione A, et al. Performance of cytology and colposcopy in diagnosis of cervical intraepithelial neoplasia (CIN) in HIV-positive and HIV-negative women. Cytopathology. 2001;12(2):84-93.

7. Chibwesha CJ, Frett B, Katundu K, Bateman AC, Shibemba A, Kapambwe S, et al. Clinical Performance Validation of 4 Point-of-Care Cervical Cancer Screening Tests in HIV-Infected Women in Zambia. Journal of Lower Genital Tract Disease.20(3):218-23.

8. Chung MH, McKenzie KP, De Vuyst H, Richardson BA, Rana F, Pamnani R, et al. Comparing Papanicolau smear, visual inspection with acetic acid and human papillomavirus cervical cancer screening methods among HIV-positive women by immune status and antiretroviral therapy. AIDS.27(18):2909-19.

9. Cohn JA, Gagnon S, Spence MR, Harrison DD, Kluzak TR, Langenberg P, et al. The role of human papillomavirus deoxyribonucleic acid assay and repeated cervical cytologic examination in the detection of cervical intraepithelial neoplasia among human immunodeficiency virus–infected women. American Journal of Obstetrics and Gynecology. 2001;184(3):322-30.

10. de Andrade AC, Luz PM, Velasque L, Veloso VG, Moreira RI, Russomano F, et al. Factors associated with colposcopy-histopathology confirmed cervical intraepithelial neoplasia among HIV-infected women from Rio De Janeiro, Brazil. PLoS ONE [Electronic Resource].6(3):e18297.

11. Delory T, Ngo-Giang-Huong N, Rangdaeng S, Chotivanich N, Limtrakul A, Putiyanun C, et al. Human Papillomavirus infection and cervical lesions in HIV infected women on antiretroviral treatment in Thailand. Journal of Infection. 2017;74(5):501-11.

12. De Vuyst H, Franceschi S, Plummer M, Mugo NR, Sakr SR, Meijer CJLM, et al. Methylation Levels of CADM1, MAL, and MIR124-2 in Cervical Scrapes for Triage of HIV-Infected, High-Risk HPV-Positive Women in Kenya. JAIDS Journal of Acquired Immune Deficiency Syndromes. 2015;70(3).

13. Duan R, Zhao X, Zhang H, Xu X, Huang L, Wu A, et al. Performance of cervical cancer screening and triage strategies among women living with HIV in China. Cancer Medicine.n/a(n/a).

14. Firnhaber C, Mayisela N, Mao L, Williams S, Swarts A, Faesen M, et al. Validation of cervical cancer screening methods in HIV positive women from Johannesburg South Africa. PLoS ONE [Electronic Resource].8(1):e53494.

15. Huchko MJ, Sneden J, Zakaras JM, Smith-McCune K, Sawaya G, Maloba M, et al. A randomized trial comparing the diagnostic accuracy of visual inspection with acetic acid to Visual Inspection with Lugol's Iodine for cervical cancer screening in HIV-infected women. PLoS ONE [Electronic Resource].10(4):e0118568.

16. Huchko MJ, Sneden J, Sawaya G, Smith-McCune K, Maloba M, Abdulrahim N, et al. Accuracy of visual inspection with acetic acid to detect cervical cancer precursors among HIV-infected women in Kenya. International Journal of Cancer.136(2):392-8.

17. Joshi S, Sankaranarayanan R, Muwonge R, Kulkarni V, Somanathan T, Divate U. Screening of cervical neoplasia in HIV-infected women in India. AIDS.27(4):607-15.

18. Kelly HA, Chikandiwa A, Sawadogo B, Gilham C, Michelow P, Lompo OG, et al. Diagnostic accuracy of cervical cancer screening and screening–triage strategies among women living with HIV-1 in Burkina Faso and South Africa: A cohort study. PLOS Medicine. 2021;18(3):e1003528.

19. Kitchener H, Nelson L, Adams J, Mesher D, Sasieni P, Cubie H, et al. Colposcopy is not necessary to assess the risk to the cervix in HIV-positive women: An international cohort study of cervical pathology in HIV-1 positive women. International Journal of Cancer. 2007;121(11):2484-91.

20. Kremer WW, van Zummeren M, Breytenbach E, Richter KL, Steenbergen RDM, Meijer CJLM, et al. The use of molecular markers for cervical screening of women living with HIV in South Africa. AIDS (London, England). 2019;33(13):2035-42.

21. Kuhn L, Saidu R, Boa R, Tergas A, Moodley J, Persing D, et al. Clinical evaluation of modifications to a human papillomavirus assay to optimise its utility for cervical cancer screening in low-resource settings: a diagnostic accuracy study. The Lancet Global Health. 2020;8(2):e296-e304.

22. Kuhn L, Wang C, Tsai WY, Wright TC, Denny L. Efficacy of human papillomavirus-based screen-and-treat for cervical cancer prevention among HIV-infected women. AIDS.24(16):2553-61.

23. Luckett R, Mogowa N, Li HJ, Erlinger A, Hacker MR, Esselen K, et al. Performance of Two-Stage Cervical Cancer Screening With Primary High-Risk Human Papillomavirus Testing in Women Living With Human Immunodeficiency Virus. Obstetrics & Gynecology.134(4):840-9.

24. Mabeya H, Khozaim K, Liu T, Orango O, Chumba D, Pisharodi L, et al. Comparison of conventional cervical cytology versus visual inspection with acetic acid among human immunodeficiency virus-infected women in Western Kenya. Journal of Lower Genital Tract Disease.16(2):92-7.

25. Maiman M, Fruchter RG, Sedlis A, Feldman J, Chen P, Burk RD, et al. Prevalence, Risk Factors, and Accuracy of Cytologic Screening for Cervical Intraepithelial Neoplasia in Women with the Human Immunodeficiency Virus. Gynecologic Oncology. 1998;68(3):233-9.

26. Mane A, Nirmalkar A, Risbud AR, Vermund SH, Mehendale SM, Sahasrabuddhe VV. HPV Genotype Distribution in Cervical Intraepithelial Neoplasia among HIV-Infected Women in Pune, India. PLOS ONE. 2012;7(6):e38731.

27. Mbulawa ZZA, Wilkin TJ, Goeieman B, Swarts A, Williams S, Levin S, et al. Xpert human papillomavirus test is a promising cervical cancer screening test for HIV-seropositive women. Papillomavirus Research.2:56-60.

28. McDonald AC, Tergas AI, Kuhn L, Denny L, Wright TC. Distribution of human papillomavirus genotypes among HIV-positive and HIV-negative women in Cape Town, South Africa. Frontiers in Oncology. 2014;4 MAR (no pagination).

29. Mungo C, Osongo CO, Ambaka J, Randa MA, Samba B, Ochieng CA, et al. Feasibility and Acceptability of Smartphone-Based Cervical Cancer Screening Among HIV-Positive Women in Western Kenya. JCO Global Oncology. 2021(7):686-93.

30. Ndizeye Z, Menon S, Van Geertruyden JP, Sauvaget C, Jacquemyn Y, Bogers JP, et al. Performance of OncoE6 TM Cervical Test in detecting cervical precancer lesions in HIV-positive women attending an HIV clinic in Bujumbura, Burundi: A cross-sectional study. BMJ Open. 2019;9(9).

31. Paboriboune P, Phongsavan K, Arounlangsy P, Flaissier B, Aphayarath O, Phimmasone P, et al. Efficacy of careHPV™ human papillomavirus screening versus conventional cytology tests for the detection of precancerous and cancerous cervical lesions among women living with HIV-1 in Lao People's Democratic Republic. Cancer Med. 2022;11(9):1984-94.

32. Petry KU, Böhmer G, Iftner T, Flemming P, Stoll M, Schmidt RE. Human Papillomavirus Testing in Primary Screening for Cervical Cancer of Human Immunodeficiency Virus-Infected Women, 1990&#x2013;1998. Gynecologic Oncology. 1999;75(3):427-31.

33. Pimple SA, Vandita P, Mishra GA, Anand KV, Saleem P, Biswas SK. Screening for early detection of cervical cancer in women living with HIV in Mumbai, India - retrospective cohort study from a tertiary cancer center. Indian Journal of Medical and Paediatric Oncology. 2022;43(1):73-83.

34. Raposo LM, Velasque L, Luz PM, Friedman RK, Cytryn A, Andrade AC, et al. [Performance by cytology and hybrid capture II in screening for high-grade squamous intraepithelial lesions in women with HIV]. Cadernos de Saude Publica.27(7):1281-91.

35. Sahasrabuddhe VV, Bhosale RA, Kavatkar AN, Nagwanshi CA, Joshi SN, Jenkins CA, et al. Comparison of visual inspection with acetic acid and cervical cytology to detect high-grade cervical neoplasia among HIV-infected women in India. International Journal of Cancer.130(1):234-40.

36. Strickler HD, Keller MJ, Hessol NA, Eltoum I-E, Einstein MH, Castle PE, et al. Primary HPV and Molecular Cervical Cancer Screening in US Women Living with HIV. Clinical Infectious Diseases. 2020.

37. Womack SD, Chirenje ZM, Gaffikin L, Blumenthal PD, McGrath JA, Chipato T, et al. HPV-based cervical cancer screening in a population at high risk for HIV infection. International Journal of Cancer.85(2):206-10.

38. Zhang HY, Fei MD, Jiang Y, Fei QY, Qian H, Xu L, et al. The diversity of human papillomavirus infection among human immunodeficiency virus-infected women in Yunnan, China. Virology Journal.11:202.

39. Zhang D-Y, Chen W, Zhang H-Y, Lv S-Q, Lu X-N, Tao Y-P, et al. Cervical neoplastic lesions in relation to CD4 T-lymphocyte counts and antiretroviral therapy among women with clinical stage 1 HIV in Yunnan, China. The Kaohsiung Journal of Medical Sciences. 2020;36(6):450-9.

40. Bansil P, Lim J, Byamugisha J, Kumakech E, Nakisige C, Jeronimo JA. Performance of Cervical Cancer Screening Techniques in HIV-Infected Women in Uganda. Journal of Lower Genital Tract Disease. 2015;19(3):215-9.

41. Firnhaber C, Mayisela N, Mao L, Williams S, Swarts A, Faesen M, et al. Validation of cervical cancer screening methods in HIV positive women from Johannesburg South Africa. PLoS ONE [Electronic Resource]. 2013;8(1):e53494.

42. Huchko MJ, Sneden J, Sawaya G, Smith-McCune K, Maloba M, Abdulrahim N, et al. Accuracy of visual inspection with acetic acid to detect cervical cancer precursors among HIV-infected women in Kenya. International Journal of Cancer. 2014;136(2):392-8.

43. Joshi S, Sankaranarayanan R, Muwonge R, Kulkarni V, Somanathan T, Divate U. Screening of cervical neoplasia in HIV-infected women in India. AIDS. 2013;27(4):607-15.

44. Sahasrabuddhe VV, Bhosale RA, Kavatkar AN, Nagwanshi CA, Joshi SN, Jenkins CA, et al. Comparison of visual inspection with acetic acid and cervical cytology to detect high-grade cervical neoplasia among HIV-infected women in India. International Journal of Cancer. 2012;130(1):234-40.

45. Segondy M, Kelly H, Magooa MP, Djigma F, Ngou J, Gilham C, et al. Performance of careHPV for detecting high-grade cervical intraepithelial neoplasia among women living with HIV-1 in Burkina Faso and South Africa: HARP study. British Journal of Cancer.115(4):425-30.

46. Anderson JR, Paramsothy P, Heilig C, Jamieson DJ, Shah K, Duerr A, et al. Accuracy of Papanicolaou Test among HIV-Infected Women. Clinical Infectious Diseases. 2006;42(4):562-8.

47. Wang R, Lee K, Gaydos CA, Anderson JR, Keller J, Coleman JS. Performance of primary self-collected human papillomavirus testing among women living with human immunodeficiency virus in the United States. Obstetrics & Gynecology. 2021;137(2):342-4.

48. Chung MH, McKenzie KP, De Vuyst H, Richardson BA, Rana F, Pamnani R, et al. Comparing Papanicolau smear, visual inspection with acetic acid and human papillomavirus cervical cancer screening methods among HIV-positive women by immune status and antiretroviral therapy. AIDS. 2013;27(18):2909-19.
